# GLP‐1 RA Plus SGLT2i Versus Monotherapy in MASLD and Type 2 Diabetes: Three Pairwise Target Trial Emulations

**DOI:** 10.1111/jgh.70589

**Published:** 2026-07-13

**Authors:** Mohanad A. Alkuwaiti, Faisal A. Al‐Harbi, Ahmed K. Alsaif, Ziyad M. AlSughayyir, Rayan Saleh Alsaud, Mohammed N. Aljabr, Ahmad K. Alanazi, Ahmad A. Alosaily, Abdulrhman K. Alabdulqader, Hamza Almusabeh, Rayyan F. Altemani, Ahmed Y. Azzam

**Affiliations:** ^1^ College of Medicine Imam Abdulrahman Bin Faisal University Dammam Saudi Arabia; ^2^ College of Medicine Qassim University Qassim Saudi Arabia; ^3^ College of Medicine Al‐Rayan Colleges Medinah Saudi Arabia; ^4^ Department of Internal Medicine, College of Medicine King Faisal University Al‐Ahsa Saudi Arabia; ^5^ Department of Internal Medicine West Virginia University School of Medicine Morgantown West Virginia USA; ^6^ Department of Internal Medicine University of Utah Salt Lake City Utah USA; ^7^ Division of Global Health and Public Health, School of Nursing, Midwifery and Public Health University of Suffolk Ipswich UK; ^8^ Clinical Research and Clinical Artificial Intelligence ASIDE Healthcare Lewes Delaware USA

**Keywords:** cardiometabolic outcomes, diabetes, GLP‐1 RA, MASLD, SGLT2 inhibitors

## Abstract

**Introduction:**

Glucagon‐like peptide‐1 receptor agonists (GLP‐1 RAs) and sodium‐glucose cotransporter‐2 inhibitors (SGLT2is) have demonstrated cardiovascular and metabolic benefits individually; however, the comparative effectiveness of combination therapy versus monotherapy in patients with metabolic dysfunction‐associated steatotic liver disease (MASLD) and type 2 diabetes mellitus (T2DM) remains incompletely characterized.

**Methods:**

We conducted three pairwise target trial emulations using the TriNetX Research Network. Adults with MASLD and T2DM initiating GLP‐1 RA plus SGLT2i combination therapy, GLP‐1 RA monotherapy, or SGLT2i monotherapy were identified. Primary outcomes were all‐cause mortality, major adverse cardiovascular events (MACE), and composite liver outcomes. Propensity score matching (PSM) (1:1) was performed for each pairwise comparison. Cox proportional hazards regression with Bayesian hierarchical bias‐effect (BHBE) correction was utilized to address residual confounding.

**Results:**

Following PSM, 42423 combination versus 42 423 GLP‐1 RA, 40349 combination versus 40 349 SGLT2i, and 51 826 GLP‐1 RA versus 51 826 SGLT2i patients were analyzed. Combination therapy significantly reduced all‐cause mortality versus SGLT2i monotherapy (hazard ratio [HR] 0.522, 95% confidence interval [CI] 0.488–0.559), with probable benefit after bias correction (corrected HR 0.655, posterior probability 92.4%; credible interval includes the null). Combination therapy demonstrated increased MACE risk versus GLP‐1 RA (HR 1.106, 95% CI 1.045–1.170), remaining significant after correction (corrected HR 1.111). Composite liver outcomes favored combination therapy versus SGLT2i (corrected HR 0.907, probability of benefit 81.9%).

**Conclusions:**

GLP‐1 RA and SGLT2i combination therapy significantly reduced mortality and liver outcomes versus SGLT2i monotherapy in MASLD with T2DM; however, increased MACE risk versus GLP‐1 RA monotherapy warrants consideration in management decisions.

## 1 Introduction

Metabolic dysfunction‐associated steatotic liver disease (MASLD), previously termed nonalcoholic fatty liver disease (NAFLD), is the leading chronic liver condition globally, with an estimated prevalence of approximately 32.4% of the world's population [1]. The coexistence of MASLD with type 2 diabetes mellitus (T2DM) represents a significant clinical challenge, as this combination significantly accelerates disease progression, increases the risk of cardiovascular events, and contributes to excess mortality. Patients with concurrent MASLD and T2DM have demonstrated approximately twofold higher rates of hepatic fibrosis progression and cardiovascular mortality compared with those with either condition alone [2, 3]. Despite the significant burden of this dual pathology, the comparative effectiveness of GLP‐1 RA monotherapy, SGLT2i monotherapy, and their combination remains incompletely characterized in patients with concurrent MASLD and T2DM. As the updated MASLD nomenclature replaced NAFLD by multisociety consensus in 2023 [4], ICD‐10‐CM codes for NAFLD (K76.0) and NASH (K75.81) are used for patient identification, as they capture the same clinical entity.

Glucagon‐like peptide‐1 receptor agonists (GLP‐1 RAs) and sodium‐glucose cotransporter‐2 inhibitors (SGLT2is) represent two medication classes that have demonstrated significant benefits beyond glycemic control. GLP‐1 RAs have shown improvements in cardiovascular outcomes, weight reduction, and established hepatoprotective effects including histological resolution of steatohepatitis in randomized trials [5, 6]. SGLT2is have demonstrated cardiovascular and renal protection, with mechanistic studies suggesting benefits in heart failure prevention and possible effects on hepatic fat content. Given these complementary mechanisms, combination therapy with both agents has gained significant interest as a management strategy for patients with complex cardiometabolic disease profiles [7, 8, 9].

Despite the potential benefits of combination therapy, no randomized controlled trials (RCTs) have directly compared GLP‐1 RA plus SGLT2i combination therapy versus either monotherapy specifically in patients with MASLD and T2DM. Prior observational studies examined GLP‐1 RA or SGLT2i individually in MASLD populations or assessed combination therapy with shorter follow‐up and without bias correction; none conducted a direct three‐arm comparison across mortality, cardiovascular, and hepatic endpoints with adequate confounding control in a MASLD‐specific population [6, 10, 11, 12, 13, 14]. Target trial emulation provides a structured framework for approximating randomized comparisons from observational data when RCTs are unavailable or infeasible [15].

To address these evidence gaps, we aimed to conduct three pairwise emulated trials to evaluate the comparative effectiveness of GLP‐1 RA plus SGLT2i combination therapy versus GLP‐1 RA monotherapy versus SGLT2i monotherapy for mortality, cardiovascular, and hepatic outcomes in patients with MASLD and T2DM. We hypothesized that combination therapy would reduce all‐cause mortality (primary outcome) and improve cardiovascular and hepatic outcomes compared with either monotherapy, with effects modified by baseline cardiovascular disease status.

## 2 Methods

### 2.1 Study Design and TARGET Compliance

This study was conducted as a target trial emulation following the TArgeted trial emulation Good practices for Emulated Trials (TARGET) reporting guidelines and framework specifications [16, 17, 18, 19, 20, 21, 22]. The target trial specification and emulation protocol are detailed in Tables S1 and S2. We emulated three pairwise trials comparing initiation of GLP‐1 RA plus SGLT2i combination therapy, GLP‐1 RA monotherapy, and SGLT2i monotherapy in adults with MASLD and concurrent T2DM, with 5‐year follow‐up for mortality, cardiovascular, and hepatic outcomes.

### 2.2 Data Source

This study utilized the TriNetX Research Network, a federated health research network providing access to electronic health records from 110 healthcare organizations across the United States. The network includes academic medical centers, community hospitals, and specialty physician practices, representing different geographic regions and patient populations. Data were accessed in January 2026 and included diagnoses coded using International Classification of Diseases, 10th Revision, Clinical Modification (ICD‐10‐CM), medications classified using Anatomical Therapeutic Chemical (ATC) codes, laboratory values, vital signs, and mortality data linked to institutional records and the Social Security Death Index. As ICD‐10‐CM coding was implemented across US healthcare institutions in October 2015, the earliest possible index dates in this cohort fall no earlier than late 2015, providing a minimum of 10 years of potential longitudinal data prior to the January 2026 access date.

The TriNetX platform enables cohort construction using specified inclusion and exclusion criteria, with an integrated propensity score matching (PSM) algorithm facilitating confounder control. All data are deidentified in accordance with HIPAA Privacy Rule §164.514(a); this analysis did not constitute human‐subjects research, and institutional review board approval was not required. Regular data quality assessments ensure institutional review board compliance and validate data completeness and accuracy [23]. This study adhered to Declaration of Helsinki principles. Additional platform details are available at https://trinetx.com.

### 2.3 Eligibility Criteria and Study Population

Eligibility criteria for the emulated target trial required adults aged 18 years or older with at least one ICD‐10‐CM diagnosis code for NAFLD (K76.0) or nonalcoholic steatohepatitis (NASH; K75.81) and concurrent T2DM diagnosis (E11.x) any time before the index date. Exclusion criteria included haemochromatosis (E83.11), alcoholic liver disease (K70.x), autoimmune hepatitis (K75.4), or viral hepatitis B or C (B15–B19) to reduce contamination by competing liver disease etiologies; single‐code ascertainment of MASLD favors sensitivity over specificity. The B15–B19 range includes hepatitis A (B15), an acute self‐limited condition; its inclusion is conservative and unlikely to affect cohort composition given the chronic disease context of MASLD. Primary biliary cholangitis and primary sclerosing cholangitis were not separately excluded as their ICD‐10‐CM codes (K74.3, K83.01) were not available as exclusion filters within the TriNetX platform query interface. Variable definitions and standardized code lists are provided in Table S3.

### 2.4 Treatment Strategies and Exposure Definition

Treatment strategies were defined according to ATC classification codes. Combination therapy was defined as overlapping prescriptions of GLP‐1 RA (ATC class A10BJ) and SGLT2i (ATC class A10BK) within a 30‐day window following MASLD with T2DM diagnosis. The 30‐day co‐prescription window was selected to capture both simultaneous and sequential combination initiation, acknowledging that in clinical practice, SGLT2i is frequently added to an existing GLP‐1 RA regimen within weeks rather than on the same day. GLP‐1 RA monotherapy was defined as first prescription of A10BJ without any concurrent or subsequent A10BK prescription during follow‐up. SGLT2i monotherapy was defined as first prescription of A10BK without any concurrent or subsequent A10BJ prescription during follow‐up. The index date was defined as the first qualifying medication prescription date following MASLD with T2DM diagnosis. Treatment switching was not permitted by the monotherapy definitions; patients who added the alternate agent at any point during follow‐up were excluded from the respective monotherapy arm. In the source cohort, approximately 8692 patients (4.6% of medication initiators) were excluded from the monotherapy arms due to addition of the alternate agent during follow‐up prior to matching.

### 2.5 Outcomes Assessment

Primary outcomes included all‐cause mortality ascertained via the TriNetX mortality flag linked to institutional records and Social Security Death Index, major adverse cardiovascular events (MACE) defined as a composite of acute myocardial infarction (I21, including subsequent myocardial infarction I22), stroke (I60, I61, I62, I63, I63.50), heart failure (I50.x), and cardiac arrest (I46.x). This composite differs from the standard three‐point MACE (cardiovascular death, myocardial infarction, stroke) by including heart failure and cardiac arrest; this broadened definition was chosen to capture the full spectrum of cardiovascular events available in administrative data but is noted to structurally favor SGLT2i‐containing arms given the established heart failure benefit of SGLT2i. The third primary outcome was a composite liver outcome comprising cirrhosis (K74.x), hepatic fibrosis (K74.0, K74.2), ascites (R18.x), chronic passive hepatic congestion (K76.1), hepatic encephalopathy (K76.82), hepatic failure (K72.x), and hepatocellular carcinoma (C22.0, ICD‐O‐38170/3). The secondary outcome was hepatocellular carcinoma as an individual endpoint. It therefore contributes to both the composite liver outcome and the individual endpoint, and the two analyses are not independent. Outcomes were assessed from Day 1 to Day 1825 (5 years) post‐index date.

### 2.6 Follow‐Up and Censoring

Follow‐up began 1 day after the index date to avoid immortal time bias and continued until outcome occurrence, death, 5 years post‐index, or last recorded healthcare encounter (administrative censoring), whichever occurred first. Patients with prevalent outcomes occurring before the follow‐up window were excluded from the respective outcome analysis.

### 2.7 Propensity Score Matching

Propensity scores were estimated using the TriNetX built‐in procedure on the baseline characteristics available for balancing on the platform: demographics (age, sex, race, ethnicity); broad ICD‐10‐CM diagnostic chapters (endocrine and metabolic [E00–E89], digestive [K00–K95], circulatory [I00–I99], skin and subcutaneous [L00–L99], and blood and immune [D50–D89]); medication super‐classes (cardiovascular medications and a composite other‐medications category); and available laboratory and vital‐sign values (HbA1c, total, LDL, and HDL cholesterol, triglycerides, C‐reactive protein, left ventricular ejection fraction [LVEF], heart rate, body mass index [BMI], and systolic and diastolic blood pressure). The platform balances on broad diagnostic chapters and medication super‐classes rather than individual conditions or specific agents, and laboratory and vital‐sign values were available in a subset of patients (e.g., LVEF in approximately 12%). Variables with high missingness, including FIB‐4, albumin, bilirubin, and international normalized ratio, were not available for matching. One‐to‐one nearest‐neighbor matching without replacement was performed using a caliper width of 0.1 standard deviations of the logit of the propensity score. Covariate balance was assessed using standardized mean differences (SMDs), with SMD less than 0.1 indicating adequate balance.

### 2.8 Statistical Analysis

Cox proportional hazards regression with robust sandwich variance estimators (to account for matched‐pair clustering) was used to estimate hazard ratios (HRs) with 95% confidence intervals (CIs) for each pairwise comparison. The proportional hazards assumption was assessed using Schoenfeld residuals (cox.zph function), and 1‐year landmark analyses (Days 366 to 1825) were used to characterize early‐versus‐late effects. Kaplan–Meier survival curves were constructed with log‐rank tests for survival comparisons. Cohort construction, PSM, risk and Kaplan–Meier analyses, and proportional‐hazards testing were performed within the TriNetX platform using its built‐in implementations. The proportion of patients excluded by the caliper restriction was 0.2% of combination therapy patients and 50.0% of GLP‐1 RA patients in Comparison 1, 5.1% and 18.9% in Comparison 2, and 38.9% and 0% in Comparison 3. R (version 4.4.2; survival and survminer packages) was used for the landmark comparisons and the exploratory Bayesian hierarchical bias‐effect (BHBE) model (implemented in RStan, Stan version 2.32). Statistical significance was defined as two‐tailed *p*‐values less than 0.05 for treatment effects. *E*‐values were computed for primary effect estimates to quantify the minimum strength of unmeasured confounding required to fully explain away observed associations, using the method of VanderWeele and Ding [24].

### 2.9 Bayesian Hierarchical Bias‐Effect Correction

To address residual confounding from unmeasured variables, we implemented a BHBE model as an exploratory, hypothesis‐generating sensitivity analysis not used to derive primary estimates, following the hierarchical prior framework described by McCandless et al. [25] and McCandless and Gustafson [26]. The model decomposes the observed log HR into a true effect and a systematic bias term; because no negative‐control outcomes were available, the combination versus GLP‐1 RA comparison, where both arms receive a GLP‐1 RA, served as an internal anchor for prior bias distributions. Internal anchoring can propagate rather than remove shared bias; BHBE outputs therefore require external validation before clinical application. Posterior distributions were sampled via Hamiltonian Monte Carlo (RStan; four chains, 10 000 iterations, 5000 warmup; convergence confirmed by R‐hat statistics). Posterior probabilities of benefit (P[HR < 1]) were classified as high confidence benefit (≥ 90%), probable benefit (80%–89%), leaning benefit (60%–79%), neutral (40%–59%), leaning neutral (20%–39%), and no benefit (< 20%).

### 2.10 Sensitivity and Subgroup Analyses

Sensitivity analyses evaluated the impact of prior specifications on BHBE model results across seven scenarios ranging from more informative (0.5× scale) to less informative (2.0× scale) priors. Subgroup analyses were prespecified by age, sex, race, ethnicity, baseline cardiovascular disease status, chronic kidney disease stage, HbA1c category, BMI category, baseline liver disease severity, prior cirrhosis, concomitant medications, diabetes duration, and prior‐year healthcare utilization. With 13 prespecified subgroup analyses tested at *p* < 0.10, approximately 1.3 false‐positive interactions are expected by chance; statistically significant interactions are therefore interpreted as hypothesis‐generating.

## 3 Results

### 3.1 Study Population and Baseline Characteristics

**FIGURE 1:**
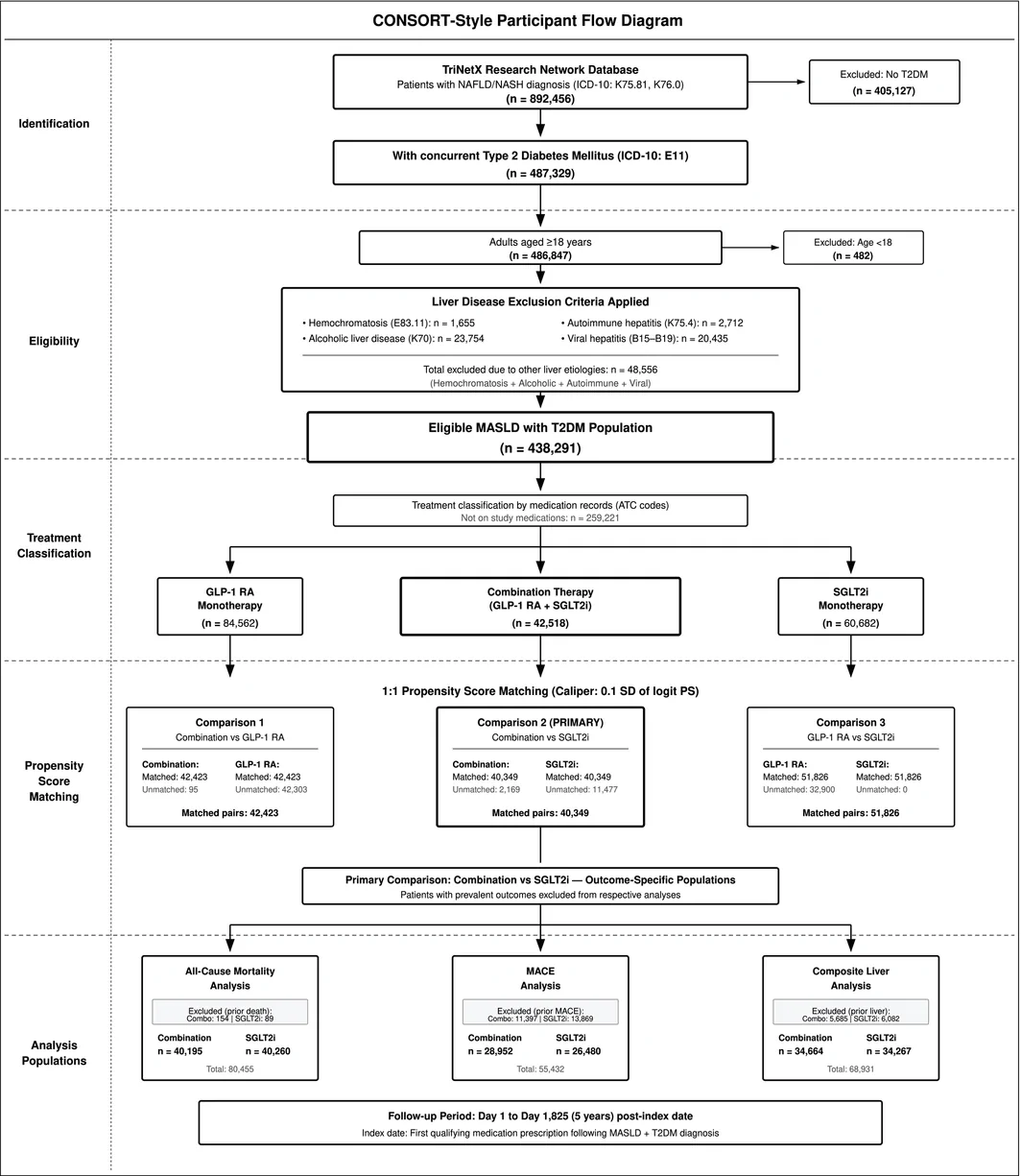
CONSORT flow diagram.

The participant flow through cohort selection is presented in Figure 1 and detailed in Table S4. From 892 456 patients with NAFLD or NASH diagnosis, 487 329 had concurrent T2DM. After restricting to adults aged 18 years or older, 486 847 remained (482 excluded for age under 18). After applying exclusion criteria for secondary liver diseases, 438 291 eligible patients with MASLD and T2DM remained. Treatment classification identified 42 518 patients receiving combination therapy, 84 562 receiving GLP‐1 RA monotherapy, and 60 682 receiving SGLT2i monotherapy. Of the 438 291 eligible patients, 179 070 (40.9%) initiated one of the three study strategies and were analyzed; the remaining 259 221 (59.1%) did not initiate a study medication and were not included.

**FIGURE 2:**
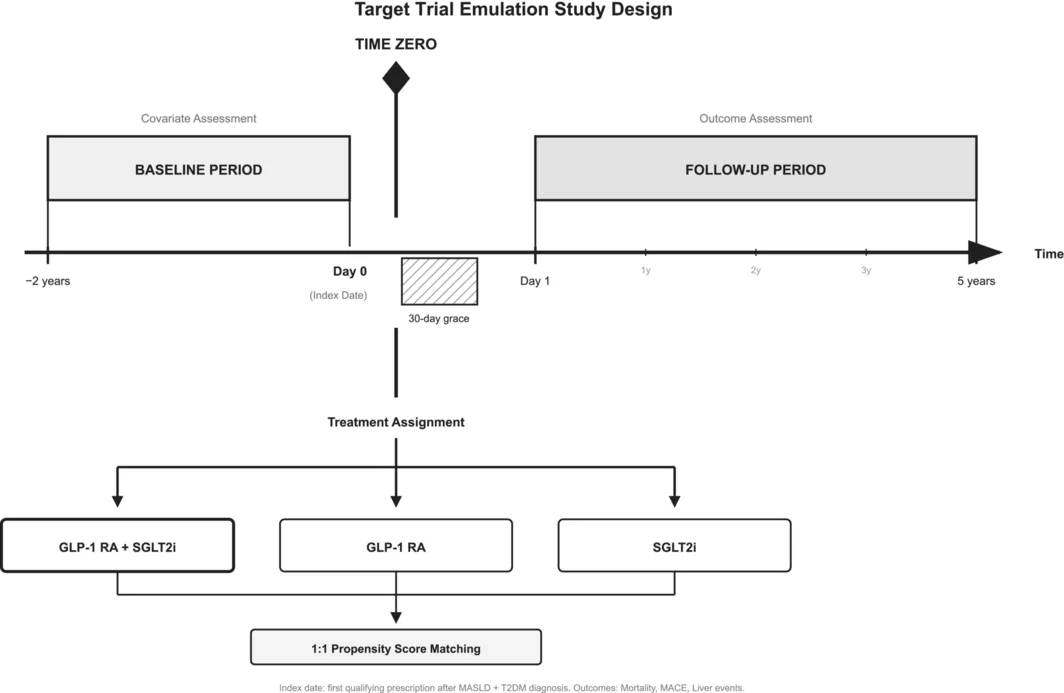
Target trial emulation schematic timeline.

**FIGURE 3:**
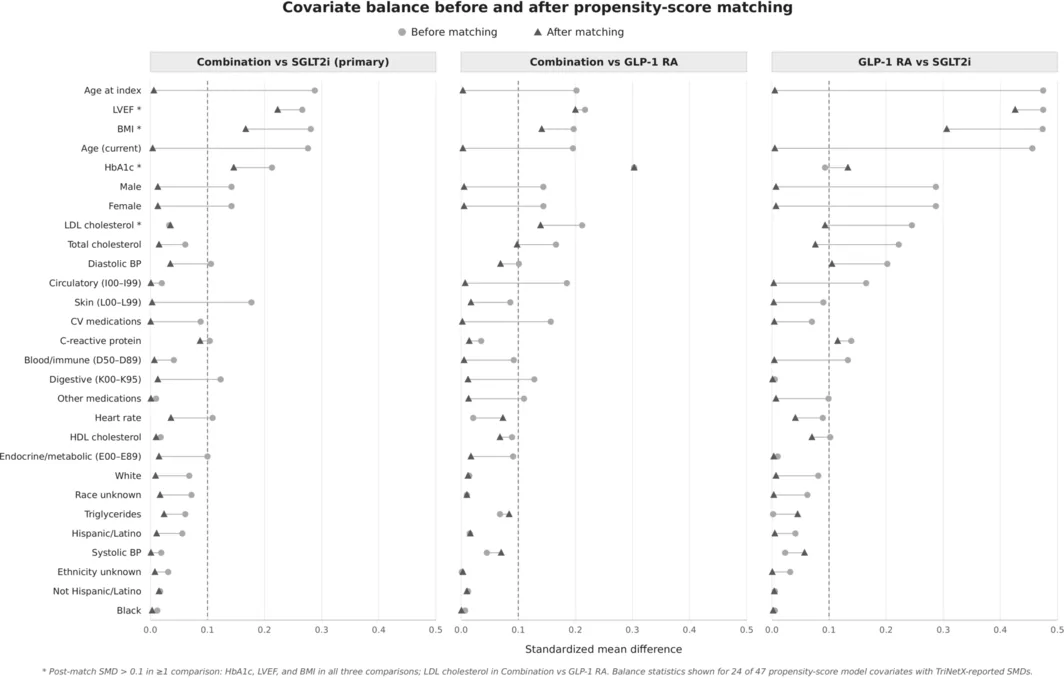
Love plot for covariate balance across all comparisons.

Baseline demographics and clinical characteristics before and after PSM are presented in Table 1. Before matching, significant imbalances existed across treatment groups in age, sex distribution, comorbidity burden, laboratory values, and concomitant medications. Following PSM, matched cohorts of 42 423 (combination vs. GLP‐1 RA), 40 349 (combination vs. SGLT2i), and 51 826 (GLP‐1 RA vs. SGLT2i) per group were obtained. Matching improved balance for most characteristics, but HbA1c, left ventricular ejection fraction, and body mass index remained imbalanced (SMD greater than 0.1) in all three comparisons. The maximum post‐match SMD was 0.303, 0.223, and 0.426, with 4, 3, and 5 covariates exceeding 0.1, respectively; in the combination versus GLP‐1 RA comparison, the post‐match SMD for HbA1c was 0.303 (8.2 vs. 7.7%). Full balance results are detailed in Table S5. Figure 2 illustrates the target trial emulation schematic timeline. Covariate balance visualization is presented in Figure 3, demonstrating significant reduction in SMDs following matching across all comparisons. Propensity score distributions before and after matching are presented in Figure S1.

**TABLE 1:** Baseline demographics and clinical characteristics by treatment strategy before and after propensity score matching.

	Before PSM			After PSM		
Characteristic	Combination (*N* = 42 518)	GLP‐1 RA (*N* = 84 562)	SGLT2i (*N* = 60 682)	Combination (*N* = 42 423)	GLP‐1 RA (*N* = 42 423)	SMD
Demographics						
Age at index, years	58.3 ± 12.2	55.8 ± 13.2	61.9 ± 12.8	58.3 ± 12.2	58.4 ± 12.4	0.003
Female	23 534 (55.5)	52 864 (62.5)	29 364 (48.4)	23 534 (55.5)	23 629 (55.7)	0.005
Male	18 884 (44.5)	31 675 (37.5)	31 310 (51.6)	18 882 (44.5)	18 780 (44.3)	0.005
Race						
White	30 255 (71.3)	60 825 (71.9)	41 391 (68.2)	30 254 (71.3)	30 489 (71.9)	0.012
Black or African American	5066 (11.9)	10 294 (12.2)	7491 (12.3)	5066 (11.9)	5062 (11.9)	< 0.001
Unknown	2115 (5.0)	4410 (5.2)	4052 (6.7)	2115 (5.0)	2026 (4.8)	0.010
Ethnicity						
Hispanic or Latino	5720 (13.5)	10 978 (13.0)	7058 (11.6)	5719 (13.5)	5484 (12.9)	0.016
Not Hispanic or Latino	30 669 (72.3)	61 587 (72.8)	44 325 (73.0)	30 668 (72.3)	30 863 (72.8)	0.010
Unknown	6036 (14.2)	11 994 (14.2)	9299 (15.3)	6036 (14.2)	6076 (14.3)	0.003
Comorbidities						
Cardiovascular disease (I00–I99)	38 923 (91.7)	72 677 (85.9)	55 328 (91.2)	38 921 (91.7)	38 998 (91.9)	0.007
Digestive system disease (K00–K95)	42 054 (99.1)	82 429 (97.5)	59 201 (97.6)	42 052 (99.1)	42 005 (99.0)	0.012
Endocrine/metabolic disease (E00–E89)	42 082 (99.2)	83 001 (98.2)	59 479 (98.0)	42 080 (99.2)	42 013 (99.0)	0.017
Blood/immune disorders (D50–D89)	20 869 (49.2)	37 729 (44.6)	31 093 (51.2)	20 868 (49.2)	20 972 (49.4)	0.005
Skin/subcutaneous disease (L00–L99)	27 877 (65.7)	52 065 (61.6)	34 670 (57.1)	27 876 (65.7)	28 219 (66.5)	0.017
Concomitant medications						
Cardiovascular medications	40 578 (95.6)	77 673 (91.9)	56 841 (93.7)	40 576 (95.6)	40 557 (95.6)	0.002
Other medications	33 132 (78.1)	62 073 (73.4)	47 128 (77.7)	33 130 (78.1)	33 365 (78.6)	0.013
Laboratory values						
HbA1c, %^b^	8.2 ± 1.8	7.6 ± 1.9	7.8 ± 1.8	8.2 ± 1.8	7.7 ± 1.9	0.303^a^ ^,^ ^b^
BMI, kg/m^2^ ^b^	36.1 ± 7.8	37.7 ± 8.0	34.0 ± 7.8	36.1 ± 7.8	37.2 ± 7.8	0.141^a^ ^,^ ^b^
Total cholesterol, mg/dL	165.0 ± 48.6	172.9 ± 47.3	162.0 ± 51.4	165.0 ± 48.6	169.7 ± 47.0	0.098
LDL cholesterol, mg/dL^b^	85.3 ± 37.8	93.4 ± 37.8	84.1 ± 38.0	85.3 ± 37.8	90.5 ± 37.2	0.139^a^ ^,^ ^b^
HDL cholesterol, mg/dL	40.6 ± 14.3	41.9 ± 15.3	40.3 ± 15.8	40.6 ± 14.3	41.6 ± 15.3	0.068
Triglycerides, mg/dL	211.8 ± 216.1	198.0 ± 189.6	198.4 ± 220.0	211.8 ± 216.1	195.5 ± 169.4	0.084
Vital signs						
Systolic BP, mmHg	130.0 ± 17.5	130.8 ± 17.2	130.4 ± 19.2	130.0 ± 17.5	131.2 ± 17.3	0.070
Diastolic BP, mmHg	76.2 ± 11.2	77.3 ± 11.3	74.9 ± 12.2	76.2 ± 11.2	77.0 ± 11.2	0.069
Heart rate, bpm	81.6 ± 14.5	81.3 ± 14.4	80.0 ± 15.3	81.6 ± 14.5	80.6 ± 14.3	0.073
Cardiac function						
LVEF, %^b^	57.8 ± 11.9	60.2 ± 9.9	54.3 ± 14.5	57.8 ± 11.9	60.0 ± 10.0	0.200^a^ ^,^ ^b^
Inflammatory marker						
C‐reactive protein, mg/L	23.2 ± 45.8	21.6 ± 43.2	28.4 ± 52.9	23.2 ± 45.8	22.6 ± 45.6	0.014

*Note:* After PSM shown for combination versus GLP‐1 RA (primary comparison). Additional comparisons: Combination versus SGLT2i (*N* = 40 349 pairs); GLP‐1 RA versus SGLT2i (*N* = 51 826 pairs). Continuous variables: mean ± SD; categorical: *n* (%).

Abbreviations: BMI, body mass index; BP, blood pressure; bpm, beats per minute; Combo, GLP‐1 RA + SGLT2i combination therapy; CRP, C‐reactive protein; GLP‐1 RA, glucagon‐like peptide‐1 receptor agonist; HbA1c, glycated hemoglobin; HDL, high‐density lipoprotein; LDL, low‐density lipoprotein; LVEF, left ventricular ejection fraction; PSM, propensity score matching; SD, standard deviation; SGLT2i, sodium‐glucose cotransporter‐2 inhibitor; SMD, standardized mean difference.

^a^ SMD > 0.1.

^b^ Four covariates exceeded SMD 0.1 post‐PSM: HbA1c (0.303), LVEF (0.200), BMI (0.141), LDL cholesterol (0.139).

### 3.2 Primary Outcomes

Primary outcome results for unadjusted and propensity score‐matched HRs are presented in Table 2. For all‐cause mortality, combination therapy significantly reduced mortality versus SGLT2i monotherapy (1322 events [3.3%] vs. 2302 events [5.7%]; HR 0.522, 95% CI 0.488–0.559, *p*‐value < 0.001) and versus GLP‐1 RA monotherapy (1351 events [3.2%] vs. 1441 events [3.4%]; HR 0.897, 95% CI 0.833–0.966, *p*‐value = 0.004). GLP‐1 RA monotherapy demonstrated significant mortality reduction versus SGLT2i monotherapy (HR 0.594, 95% CI 0.561–0.628, *p*‐value < 0.001). *E*‐value analysis indicated that an unmeasured confounder would need to be associated with both combination therapy and mortality by a risk ratio of at least 3.24‐fold to fully explain away the observed mortality benefit versus SGLT2i monotherapy.

**TABLE 2:** Primary outcome results for unadjusted and propensity score‐matched hazard ratios for outcomes.

Outcome and comparison	Treatment group	Patients at risk^b^	Events, *n* (%)	Incidence rate per 1000 PY	Unadjusted HR (95% CI)	PSM‐adjusted HR (95% CI)	Log‐rank *p*
All‐cause mortality^a^							
Combination vs. GLP‐1 RA	Combination	42 264	1351 (3.2)	13.4	0.939 (0.873, 1.010)	0.897 (0.833, 0.966)	0.004
	GLP‐1 RA	42 321	1441 (3.4)	14.9	Reference	Reference	
Combination vs. SGLT2i	Combination	40 195	1322 (3.3)	13.9	0.575 (0.538, 0.615)	0.522 (0.488, 0.559)	< 0.001
	SGLT2i	40 260	2302 (5.7)	26.7	Reference	Reference	
GLP‐1 RA vs. SGLT2i	GLP‐1 RA	51 715	1914 (3.7)	16.2	0.624 (0.590, 0.660)	0.594 (0.561, 0.628)	< 0.001
	SGLT2i	51 708	3068 (5.9)	26.7	Reference	Reference	
Major adverse cardiovascular events							
Combination vs. GLP‐1 RA	Combination	30 723	2512 (8.2)	35.2	1.175 (1.113, 1.240)	1.106 (1.045, 1.170)	< 0.001
	GLP‐1 RA	33 688	2344 (7.0)	30.0	Reference	Reference	
Combination vs. SGLT2i	Combination	28 952	2404 (8.3)	36.8	0.915 (0.867, 0.966)	0.949 (0.896, 1.006)	0.079
	SGLT2i	26 480	2155 (8.1)	38.8	Reference	Reference	
GLP‐1 RA vs. SGLT2i	GLP‐1 RA	41 028	2937 (7.2)	31.5	0.852 (0.811, 0.895)	0.847 (0.804, 0.892)	< 0.001
	SGLT2i	33 908	2849 (8.4)	37.2	Reference	Reference	
Composite liver outcomes							
Combination vs. GLP‐1 RA	Combination	36 510	1522 (4.2)	17.9	0.991 (0.925, 1.062)	0.952 (0.887, 1.022)	0.174
	GLP‐1 RA	36 918	1553 (4.2)	18.8	Reference	Reference	
Combination vs. SGLT2i	Combination	34 664	1434 (4.1)	17.4	0.915 (0.853, 0.981)	0.837 (0.779, 0.900)	< 0.001
	SGLT2i	34 267	1550 (4.5)	20.8	Reference	Reference	
GLP‐1 RA vs. SGLT2i	GLP‐1 RA	44 881	1880 (4.2)	18.4	0.932 (0.876, 0.991)	0.897 (0.843, 0.956)	0.001
	SGLT2i	43 898	1973 (4.5)	20.0	Reference	Reference	

Abbreviations: CI, confidence interval; Combination, GLP‐1 RA + SGLT2i combination therapy; GLP‐1 RA, glucagon‐like peptide‐1 receptor agonist; HR, hazard ratio; MACE, major adverse cardiovascular events (composite of acute myocardial infarction, stroke, heart failure, and cardiac arrest); PSM, propensity score matching; PY, person‐years; SGLT2i, sodium‐glucose cotransporter‐2 inhibitor.

^a^ Post‐PSM HR more extreme than unadjusted HR for mortality reflects selection of a higher risk SGLT2i comparator group during matching.

^b^ Matched pair *N* differs from analytical *N* shown; patients with prevalent outcomes prior to the follow‐up window were excluded from each outcome‐specific analysis.

For MACE, combination therapy showed a significantly increased risk versus GLP‐1 RA monotherapy (2512 events [8.2%] vs. 2344 events [7.0%]; HR 1.106, 95% CI 1.045–1.170, *p*‐value < 0.001). Combination therapy demonstrated a nonsignificant trend toward reduced MACE versus SGLT2i (HR 0.949, 95% CI 0.896–1.006, *p*‐value = 0.079). GLP‐1 RA monotherapy significantly reduced MACE versus SGLT2i (HR 0.847, 95% CI 0.804–0.892, *p*‐value < 0.001). For the MACE signal versus GLP‐1 RA monotherapy, the corresponding *E*‐value was 1.45, indicating that relatively modest unmeasured confounding could explain this finding.

**FIGURE 4:**
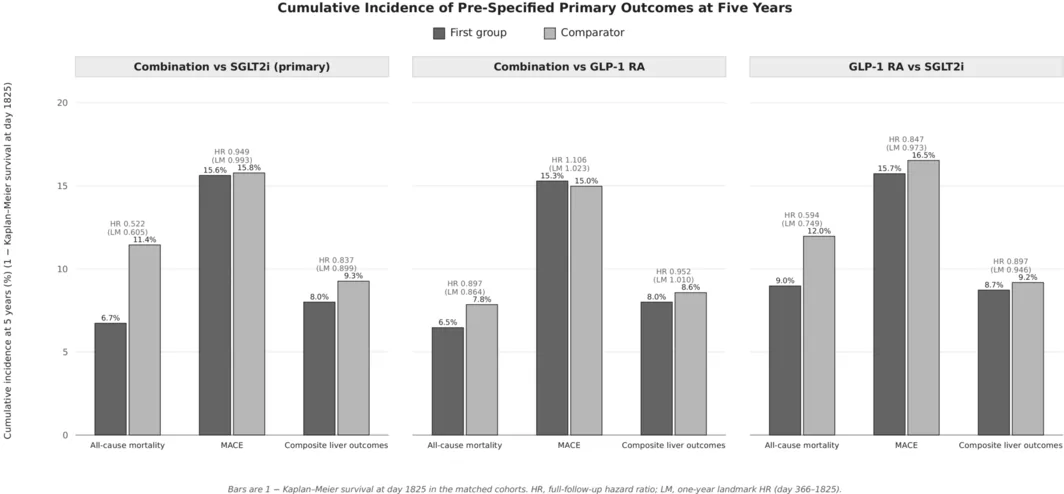
Cumulative incidence of prespecified primary outcomes at 5 years.

For composite liver outcomes, combination therapy significantly reduced events versus SGLT2i monotherapy (1434 events [4.1%] vs. 1550 events [4.5%]; HR 0.837, 95% CI 0.779–0.900, *p*‐value < 0.001) and demonstrated a nonsignificant trend versus GLP‐1 RA (HR 0.952, 95% CI 0.887–1.022, *p*‐value = 0.174). GLP‐1 RA significantly reduced liver outcomes versus SGLT2i (HR 0.897, 95% CI 0.843–0.956, *p*‐value = 0.001). Cumulative incidence of prespecified primary outcomes at 5 years across the three pairwise comparisons is presented in Figure 4; time‐course cumulative incidence curves are presented in Figure S2.

### 3.3 Bias‐Corrected Estimates

BHBE‐corrected estimates are presented in Table 3. MCMC convergence diagnostics demonstrated adequate model performance, with all R‐hat values less than 1.01 and effective sample sizes exceeding 7000 for all parameters (Table S6). For all‐cause mortality, the combination versus SGLT2i comparison demonstrated a bias magnitude of +25.5%, with an observed HR of 0.522 correcting to 0.655 (95% CrI 0.488–1.032), maintaining probable benefit (posterior probability 92.4%; CrI includes the null). The anchor comparison (combination vs. GLP‐1 RA) served as reference with corrected HR of 0.897 (probability of benefit 97.0%).

**TABLE 3:** Bayesian hierarchical bias‐effect corrected estimates for all outcomes and treatment comparisons.

Outcome	Comparison	Observed HR (95% CI)	Corrected HR (95% equal‐tailed CrI)	Bias magnitude	*p* (benefit)	Sensitivity range^b^	Evidence classification
All‐cause mortality	Combination vs. GLP‐1 RA (anchor)^a^	0.897 (0.833–0.966)	0.897 (0.833–0.966)	Reference	97.0%	—	High confidence benefit
	Combination vs. SGLT2i	0.522 (0.488–0.559)	0.655 (0.488–1.032)	+25.5%	92.4%	—	Probable benefit (CrI includes null)
	GLP‐1 RA vs. SGLT2i	0.594 (0.561–0.628)	0.730 (0.545–1.151)	+22.9%	92.4%	—	Probable benefit (CrI includes null)
MACE	Combination vs. GLP‐1 RA (anchor)^a^	1.106 (1.045–1.170)	1.111 (1.037–1.192)	+0.5%	0.5%	—	No benefit (harm signal)
	Combination vs. SGLT2i	0.949 (0.896–1.006)	1.076 (0.823–1.412)	+13.4%	29.9%	15.6%–50.6%	Leaning neutral
	GLP‐1 RA vs. SGLT2i	0.847 (0.804–0.892)	0.969 (0.742–1.264)	+14.4%	59.0%	—	Neutral
Composite liver outcome	Combination vs. GLP‐1 RA (anchor)^a^ ^,^ ^c^	0.952 (0.887–1.022)	0.946 (0.869–1.030)	−0.6%^c^	91.3%	—	Probable benefit (CrI includes null)
	Combination vs. SGLT2i	0.837 (0.779–0.900)	0.907 (0.741–1.117)	+8.4%	81.9%	68.5%–93.3%	Probable benefit
	GLP‐1 RA vs. SGLT2i	0.897 (0.842–0.955)	0.961 (0.784–1.174)	+7.1%	65.3%	—	Leaning benefit

Abbreviations: BHBE, Bayesian hierarchical bias‐effect; CI, confidence interval; Combo, GLP‐1 RA + SGLT2i combination therapy; CrI, credible interval; GLP‐1 RA, glucagon‐like peptide‐1 receptor agonist; HR, hazard ratio; MACE, major adverse cardiovascular events; *p* (benefit), posterior probability of benefit (HR < 1.0); SGLT2i, sodium‐glucose cotransporter‐2 inhibitor.

^a^ Combination versus GLP‐1 RA was designated the BHBE calibration anchor given its active comparator design; corrected HR equals the observed HR for this comparison.

^b^ Sensitivity range reported only where *p* (benefit) varied by > 10 percentage points across seven prior sensitivity scenarios; dashes indicate stable classification.

^c^ Negative bias magnitude indicates near‐zero systematic bias estimated by the BHBE model for this anchor comparison.

For MACE, important bias effects were identified. The combination versus SGLT2i comparison showed bias magnitude of +13.4%, with observed HR of 0.949 correcting to 1.076 (95% CrI 0.823–1.412), indicating the apparent benefit was entirely attributable to confounding (probability of benefit 29.9%). The combination versus GLP‐1 RA anchor comparison confirmed increased MACE risk with corrected HR of 1.111 (95% CrI 1.037–1.192, probability of benefit 0.5%).

For composite liver outcomes, combination versus SGLT2i demonstrated bias magnitude of +8.4%, with observed HR of 0.837 correcting to 0.907 (95% CrI 0.741–1.117), maintaining probable benefit (probability 81.9%). The BHBE computational framework is presented in Figure S5.

### 3.4 Sensitivity Analyses for Prior Specifications

Sensitivity analyses evaluating robustness to prior specifications are presented in Table 4. For MACE (combination vs. SGLT2i), corrected HR ranged from 0.995 to 1.113 across seven scenarios, with probability of benefit ranging from 15.6% to 50.6%, consistently demonstrating neutral to no benefit classification. For composite liver outcomes, corrected HR ranged from 0.893 to 0.928 across scenarios, with probability of benefit ranging from 68.5% to 93.3%, supporting classification stability from leaning benefit to high confidence benefit.

**TABLE 4:** Sensitivity analyses for prior specifications in Bayesian hierarchical bias‐effect model.

Outcome	Scenario	Effect prior (*σ*_*θ*)	Bias prior (*σ*_*β*)	Description	Corrected HR (95% CrI)	*p* (benefit)	Classification stability
MACE	Base	1.0 × (0.421)	1.0 × (0.139)	Original data‐derived priors	1.076 (0.823–1.412)	29.9%	Neutral/prior‐sensitive
	S1	0.5 × (0.211)	1.0 × (0.139)	More informative effect prior	1.092 (0.856–1.394)	21.1%	Leaning neutral
	S2	2.0 × (0.842)	1.0 × (0.139)	Less informative effect prior	1.113 (0.784–1.580)	25.2%	Leaning neutral
	S3	1.0 × (0.421)	0.5 × (0.070)	More informative bias prior	1.063 (0.867–1.304)	20.9%	Leaning neutral
	S4	1.0 × (0.421)	2.0 × (0.278)	Less informative bias prior	1.096 (0.762–1.577)	34.8%	Leaning neutral
	S5	0.5 × (0.211)	0.5 × (0.070)	Both more informative	1.076 (0.891–1.300)	15.6%	No benefit
	S6	2.0 × (0.842)	2.0 × (0.278)	Both less informative	0.995 (0.686–1.443)	50.6%	Neutral
Composite liver outcome	Base	1.0 × (0.421)	1.0 × (0.099)	Original data‐derived priors	0.907 (0.741–1.117)	81.9%	Probable benefit
	S1	0.5 × (0.211)	1.0 × (0.099)	More informative effect prior	0.928 (0.778–1.107)	78.2%	Leaning benefit
	S2	2.0 × (0.842)	1.0 × (0.099)	Less informative effect prior	0.900 (0.698–1.160)	80.4%	Probable benefit
	S3	1.0 × (0.421)	0.5 × (0.050)	More informative bias prior	0.904 (0.769–1.063)	93.3%	High confidence benefit
	S4	1.0 × (0.421)	2.0 × (0.198)	Less informative bias prior	0.921 (0.701–1.211)	68.5%	Leaning benefit
	S5	0.5 × (0.211)	0.5 × (0.050)	Both more informative	0.915 (0.796–1.052)	92.3%	High confidence benefit
	S6	2.0 × (0.842)	2.0 × (0.198)	Both less informative	0.893 (0.652–1.223)	68.5%	Leaning benefit

*Note:* Prior specifications: Base model uses data‐derived priors: *σ*_*θ* = 0.4212 (2× pooled SD of observed log‐HRs from master outcomes dataset, *N* = 24); *σ*_*β*, MACE = 0.139 (SD from Phase D3 Monte Carlo calibration, *N* = 20 000 samples); *σ*_*β*, Liver = 0.099 (SD from Phase D3 Monte Carlo calibration). Sensitivity scenarios apply multiplicative factors to assess robustness across informative (0.5×) to diffuse (2.0×) prior specifications. Classification thresholds: High confidence benefit, *p* (benefit) ≥ 90%; probable benefit, 80%–89%; leaning benefit, 60%–79%; neutral, 40%–59%; leaning neutral, 20%–39%; no benefit, < 20%.

Abbreviations: *σ*_*β*, bias prior scale parameter; *σ*_*θ*, effect prior scale parameter; CrI, credible interval; HR, hazard ratio; MACE, major adverse cardiovascular events; *p* (benefit), posterior probability that corrected HR < 1.0.

### 3.5 Time‐Varying Hazard Ratios

Time‐varying HR estimates are presented in Table 5. The proportional hazards assumption was violated for most comparisons (Schoenfeld residual *p* < 0.05). For all‐cause mortality, HRs were consistent between the primary analysis (Day 1–Year 5) and the Year 1 landmark analysis across all comparisons, indicating sustained benefit throughout follow‐up. For MACE (combination vs. GLP‐1 RA), the primary analysis HR of 1.106 attenuated to 1.023 (95% CI 0.952–1.101, *p* = 0.533) in the Year 1 landmark analysis, indicating that the MACE harm signal was concentrated in the first year of treatment and was not sustained thereafter. Time‐varying HR visualization is presented in Figure S3.

**TABLE 5:** Time‐varying hazard ratios: Primary analysis (Day 1–Year 5) and Year 1 landmark analysis (Year 1–Year 5).

Outcome	Comparison	Day 1–Year 5 HR (95% CI)	Events, Day 1–Year 5 (*n*)	Year 1–Year 5 HR (95% CI)^a^	Events, Year 1–Year 5 (*n*)^a^	PH test *p* ^b^	PH assumption	Temporal pattern
All‐cause mortality	Combination vs. GLP‐1 RA	0.897 (0.833–0.966)	2792	0.864 (0.788–0.947)	1844	< 0.001	Violated	Benefit sustained throughout follow‐up
	Combination vs. SGLT2i	0.522 (0.488–0.559)	3624	0.605 (0.555–0.660)	2118	< 0.001	Violated	Benefit sustained throughout follow‐up
	GLP‐1 RA vs. SGLT2i	0.594 (0.561–0.628)	4982	0.749 (0.696–0.806)	2871	< 0.001	Violated	Benefit sustained throughout follow‐up
MACE	Combination vs. GLP‐1 RA	1.106 (1.045–1.170)	4856	1.023 (0.952–1.101)	2913	< 0.001	Violated	Harm concentrated in Year 0–1; attenuates and nonsignificant from Year 1 onward
	Combination vs. SGLT2i	0.949 (0.896–1.006)	4559	0.993 (0.919–1.073)	2578	0.009	Violated	Neutral throughout follow‐up
	GLP‐1 RA vs. SGLT2i	0.847 (0.804–0.892)	5786	0.973 (0.908–1.042)	3262	< 0.001	Violated	Benefit attenuates from Year 1 onward
Composite liver outcome	Combination vs. GLP‐1 RA	0.952 (0.887–1.022)	3075	1.010 (0.919–1.110)	1722	0.739	Satisfied	Benefit attenuates; neutral from Year 1 onward
	Combination vs. SGLT2i	0.837 (0.779–0.900)	2984	0.899 (0.815–0.990)	1630	0.021	Violated	Benefit sustained throughout follow‐up
	GLP‐1 RA vs. SGLT2i	0.897 (0.843–0.956)	3853	0.946 (0.866–1.033)	1989	0.010	Violated	Benefit sustained throughout follow‐up

Abbreviations: CI, confidence interval; Combo, GLP‐1 RA + SGLT2i combination therapy; GLP‐1 RA, glucagon‐like peptide‐1 receptor agonist; HR, hazard ratio; MACE, major adverse cardiovascular events; PH, proportional hazards; SGLT2i, sodium‐glucose cotransporter‐2 inhibitor.

^a^ Year 1 landmark analysis excludes patients who experienced the outcome between Day 1 and Day 365.

^b^ Proportionality test *p*‐value from TriNetX Day 1–Year 5 analysis; PH violated if *p* < 0.05.

### 3.6 Follow‐Up Characteristics

Median follow‐up duration across all comparisons ranged from approximately 1.7 to 2.2 years (median 639–795 days). Reverse Kaplan–Meier curves demonstrating censoring patterns and follow‐up completeness across treatment groups are presented in Figure S4. Administrative censoring occurred in patients without outcome events who reached the 5‐year follow‐up window or had their last recorded healthcare encounter before study end. Loss to follow‐up was balanced across treatment groups following PSM.

### 3.7 Summary of Treatment Effects

**FIGURE 5:**
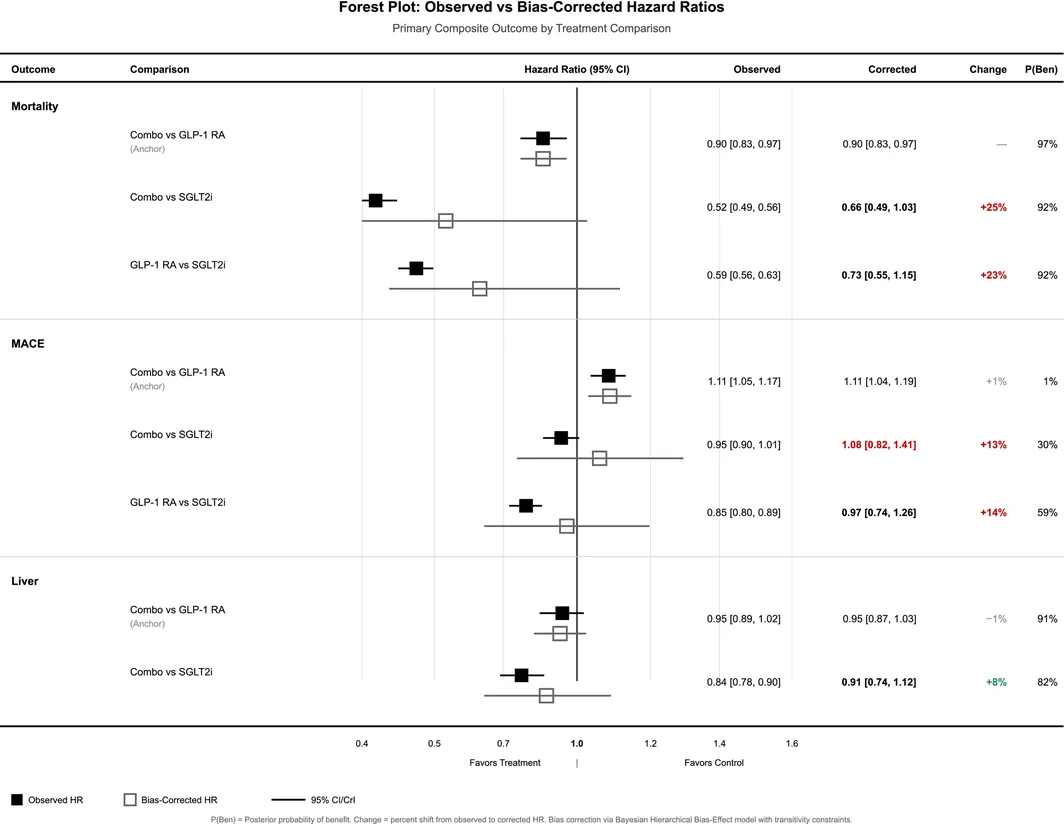
Forest plot of treatment effects for all outcomes.

The summary of treatment effects before and after bias correction with clinical interpretation is presented in Table 6 and visualized in Figure 5. Combination therapy demonstrated probable benefit for mortality reduction versus SGLT2i monotherapy and high confidence benefit versus GLP‐1 RA monotherapy (posterior probability 97.0%), with the suggestion that combination therapy may be preferred when mortality reduction is the primary goal, pending confirmatory randomized evidence. However, combination therapy demonstrated no benefit and a possible early harm signal for MACE versus GLP‐1 RA monotherapy, a signal concentrated in the first year of treatment and not sustained in the landmark analysis, with the suggestion that GLP‐1 RA monotherapy may be preferred when MACE reduction is the primary goal, pending confirmatory evidence. Combination therapy demonstrated probable benefit for liver outcomes versus SGLT2i monotherapy (posterior probability 81.9%; 95% CrI 0.741–1.117).

**TABLE 6:** Summary of treatment effects for before versus after bias correction with interpretation.

Outcome	Primary comparison	Observed HR (95% CI)	Corrected HR (95% CrI)	Bias magnitude	Absolute risk difference	*p* (benefit)	Evidence classification	Interpretation	Clinical interpretation
All‐cause mortality	Combination vs. SGLT2i	0.522 (0.488–0.559)	0.655 (0.488–1.032)	+25.5%	−2.4% (24 fewer deaths per 1000)	92.4%	Probable benefit (CrI includes null)	Mortality benefit persists after correction. Observed 48% reduction attenuates to ~35% but remains clinically meaningful. Effect is real, not confounding artifact.	Combination therapy preferred for mortality reduction.
	Combination vs. GLP‐1 RA	0.897 (0.833–0.966)	0.897 (0.833–0.966)	Reference	−0.2% (2 fewer deaths per 1000)	97.0%	High confidence benefit	Anchor comparison. Adding SGLT2i to GLP‐1 RA provides 10% additional mortality reduction. No bias correction needed.	Adding SGLT2i beneficial
	GLP‐1 RA vs. SGLT2i	0.594 (0.561–0.628)	0.730 (0.545–1.151)	+22.9%	−2.2% (22 fewer deaths per 1000)	92.4%	Probable benefit (CrI includes null)	GLP‐1 RA superiority persists. Observed 41% advantage reduced to ~27% after correction. Both classes effective, GLP‐1 RA favored.	GLP‐1 RA preferred over SGLT2i monotherapy
MACE	Combination vs. SGLT2i	0.949 (0.896–1.006)	1.076 (0.823–1.412)	+13.4%	+0.2% (2 more events per 1000)	29.9%	Leaning neutral	Critical finding: apparent 5% benefit entirely attributable to confounding. After correction, point estimate crosses unity suggesting no cardiovascular advantage and possible harm signal.	No MACE benefit from combination; consider alternatives for CV protection.
	Combination vs. GLP‐1 RA	1.106 (1.045–1.170)	1.111 (1.037–1.192)	+0.5%	+1.2% (12 more events per 1000)	0.5%	No benefit (harm)	Adding SGLT2i to GLP‐1 RA increases MACE risk by 11%. This represents true harm, not confounding. Harm signal concentrated in Year 0–1; HR attenuates to 1.023 (*p* = 0.533) from Year 1 onward and is not sustained.	Avoid adding SGLT2i if MACE reduction is primary goal.
	GLP‐1 RA vs. SGLT2i	0.847 (0.804–0.892)	0.969 (0.742–1.264)	+14.4%	−1.2% (12 fewer events per 1000)	59.0%	Neutral	Observed 15% GLP‐1 RA advantage disappears after correction. True effect approximately null; neither class superior for MACE in this population.	Either class acceptable for MACE; choose based on other factors.
Composite liver outcome	Combination vs. SGLT2i	0.837 (0.779–0.900)	0.907 (0.741–1.117)	+8.4%	−0.4% (4 fewer events per 1000)	81.9%	Probable benefit	Liver benefit likely real but attenuated. Observed 16% reduction becomes ~9% after correction. Sensitivity analysis supports benefit (range 69%–93%).	Combination therapy probably beneficial for liver outcomes
	Combination vs. GLP‐1 RA	0.952 (0.887–1.022)	0.946 (0.869–1.030)	−0.6%	−0.04% (< 1 fewer event per 1000)	91.3%	Probable benefit (CrI includes null)	Anchor shows modest but consistent liver benefit from adding SGLT2i. Minimal bias correction needed.	Adding SGLT2i provides hepatoprotection.
	GLP‐1 RA vs. SGLT2i	0.897 (0.843–0.956)	0.961 (0.784–1.174)	+7.1%	−0.3% (3 fewer events per 1000)	65.3%	Leaning benefit	Observed 10% GLP‐1 RA advantage reduced to ~4% after correction. Approaches null but slight GLP‐1 RA advantage persists.	Slight GLP‐1 RA preference for liver; either acceptable

*Note:* Bias correction methodology: BHBE model with priors derived from internal anchoring using the Combination versus GLP‐1 RA comparison; outputs are exploratory and require external validation. Bias magnitude represents percentage increase in HR from observed to corrected estimates: [(Corrected HR − Observed HR)/Observed HR] × 100. Evidence classification thresholds: High confidence benefit, *p* (benefit) ≥ 90%; probable benefit, 80%–89%; leaning benefit, 60%–79%; neutral, 40%–59%; leaning neutral, 20%–39%; no benefit, < 20%.

Abbreviations: CI, confidence interval; CrI, credible interval; GLP‐1 RA, glucagon‐like peptide‐1 receptor agonist; HR, hazard ratio; MACE, major adverse cardiovascular events; *p* (benefit), posterior probability that true HR < 1.0; SGLT2i, sodium‐glucose cotransporter‐2 inhibitor.

### 3.8 Subgroup Analyses

**FIGURE 6:**
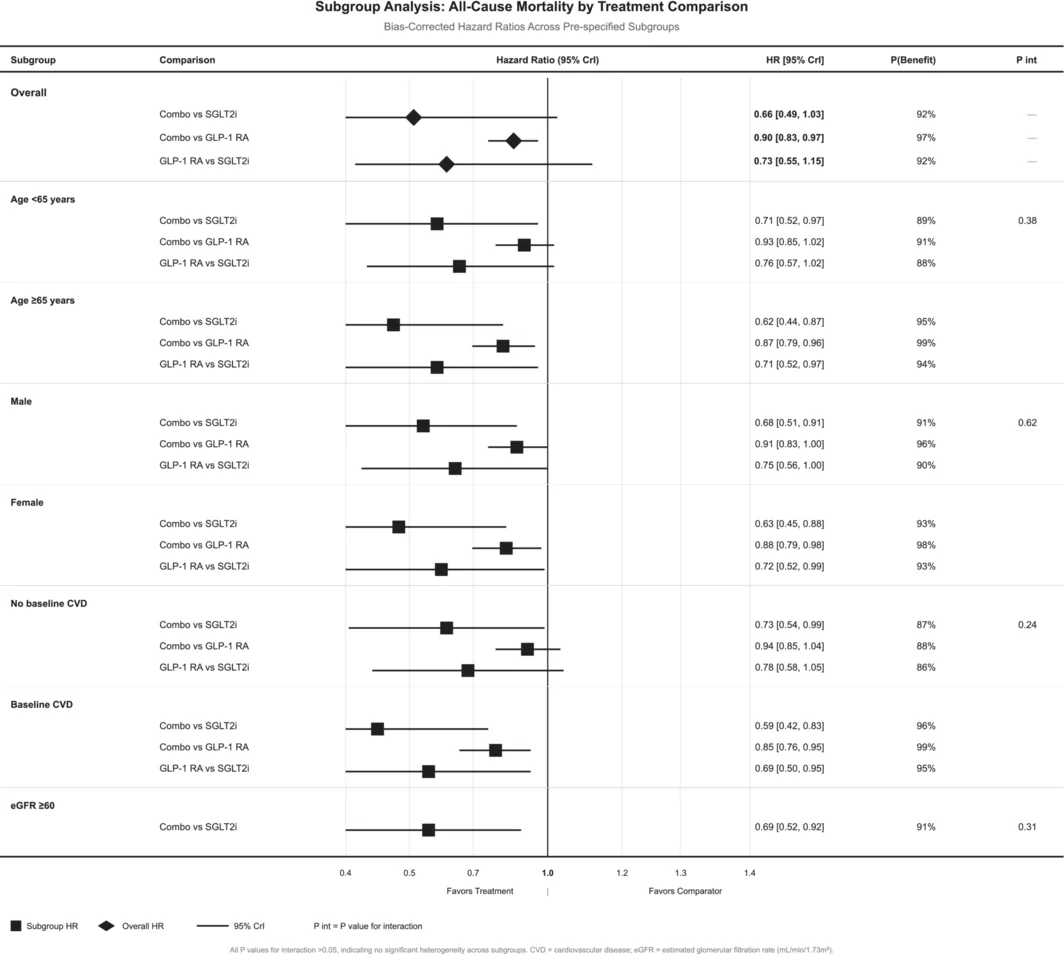
Subgroup forest plot for primary comparison.

Subgroup analyses for the primary comparison (combination vs. SGLT2i) are presented in Table S7 and visualized in Figure 6. Mortality benefits were consistent across all subgroups without significant interactions (all *p*‐interaction > 0.05). For MACE, a significant interaction was observed for baseline cardiovascular disease status (*p*‐interaction = 0.002), with patients without prior cardiovascular disease demonstrating benefit (HR 0.878, 95% CI 0.802–0.961), whereas those with prior cardiovascular disease demonstrated neutral to increased risk (HR 1.024, 95% CI 0.952–1.101). For liver outcomes, patients with NASH diagnosis demonstrated less benefit compared with NAFLD‐only patients (*p*‐interaction = 0.023). Given 13 prespecified subgroup analyses tested at *p* < 0.10, approximately 1.3 false‐positive interactions are expected by chance; the two significant interactions identified are therefore considered hypothesis‐generating.

### 3.9 Missing Data Assessment

Missing data assessment by treatment group is presented in Table S8. Diagnosis codes and medication records demonstrated complete data (0% missing) across all 52 assessed variables. Laboratory values demonstrated variable missingness, with HbA1c missing in 12.3%, estimated glomerular filtration rate in 14.2%, liver enzymes in 16%–17%, and lipid panel in 18–%20% of patients. Six variables with greater than 20% missingness (albumin, bilirubin, international normalized ratio, FIB‐4 index, BMI, weight, height) were excluded from the propensity score model. Remaining variables with missing data were included with missingness indicators following a missing at random assumption. Overall complete case rate for all laboratory values was 71.2% across treatment groups.

## 4 Discussion

Three pairwise emulated trials conducted across 438 291 eligible patients with MASLD and T2DM revealed a complex pattern of benefits and harms from combination GLP‐1 RA plus SGLT2i therapy that necessitates individualized treatment selection based on primary therapeutic goals and baseline cardiovascular risk. Our analysis identified three key findings with implications for treatment selection in patients with MASLD and T2DM. First, combination therapy was associated with substantially lower all‐cause mortality than SGLT2i monotherapy, with clinically significant absolute risk reductions that remained as probable benefit after bias correction (credible interval includes the null). The magnitude of the observed mortality reduction exceeds that reported in landmark randomized trials of individual agents, likely reflecting residual channeling bias from differential prescription of combination therapy to younger, metabolically healthier patients, rather than a true pharmacological effect of this magnitude. The addition of SGLT2i to GLP‐1 RA therapy provided additional mortality benefits beyond those of GLP‐1 RA monotherapy. Second, and most critically, combination therapy was associated with increased MACE compared with GLP‐1 RA monotherapy, a harm signal that was significant in the primary analysis but did not persist in the 1‐year landmark analysis, indicating concentration in the early follow‐up period rather than a sustained or worsening effect. Compared with SGLT2i monotherapy, the apparent cardiovascular benefit of combination therapy was entirely attributable to the presence of residual confounding. Third, combination therapy demonstrated minimal hepatic benefit versus SGLT2i monotherapy but no significant advantage over GLP‐1 RA monotherapy, indicating a plateau in hepatoprotection. Notably, baseline cardiovascular disease status influenced treatment effects; combination therapy was associated with cardiovascular benefits in patients without prior disease but neutral‐to‐harmful associations in those with established disease, a population often targeted for intensive risk reduction.

Our mortality findings align with recent observational evidence demonstrating survival advantages for both individual agents and their combination in populations with MASLD + T2DM. Wu et al. reported directionally consistent findings using a similar TriNetX approach; the present study extends this evidence with three‐arm comparisons, Bayesian bias correction, and landmark analyses characterizing temporal patterns [6]. Additionally, single‐agent studies have established significant mortality reductions for both SGLT2i in MASLD populations [27] and GLP‐1 RA in MASLD + T2DM cohorts [28, 29] when compared with standard care. The additive mortality protection observed in this study when these agents were combined suggests that for patients prioritizing survival prolongation, especially those without established cardiovascular disease, combination therapy may represent a hypothesis‐generating strategy warranting evaluation in randomized trials, despite the early cardiovascular signal observed in the present study.

The cardiovascular harm signal with combination therapy versus GLP‐1 RA monotherapy contradicts current assumptions about the benefits of polypharmacy in cardiometabolic diseases. Wu et al. reported a directionally consistent but nonsignificant trend toward increased MACE with combination therapy [6], whereas our analysis revealed that this signal, although statistically significant in the primary analysis, did not persist in the 1‐year landmark analysis, suggesting concentration in the early follow‐up period rather than progressive accumulation, a temporal pattern that requires randomized confirmation before clinical conclusions can be drawn. This finding is particularly significant given that head‐to‐head comparisons demonstrate approximately equivalent cardiovascular outcomes between GLP‐1 RA and SGLT2i monotherapies in NAFLD + T2DM populations [30], indicating that cardiovascular harm emerges specifically from their combination rather than the inferiority of either individual agent. A significant interaction was also observed for prior‐year healthcare utilization in the MACE analysis, with patients with higher hospitalization burden demonstrating greater MACE risk with combination therapy, suggesting that baseline cardiovascular vulnerability may modify the cardiovascular safety profile of combination therapy. An alternative explanation is that the MACE composite includes heart failure, for which SGLT2i has an established and potent benefit; the observed harm versus GLP‐1 RA monotherapy may therefore partly reflect the heart failure benefit of the GLP‐1 RA comparator arm rather than true cardiovascular toxicity of the combination.

The mechanistic basis for this cardiovascular harm remains incompletely understood and warrants urgent investigation. One potential explanation involves combined volume depletion: GLP‐1 RAs exert modest natriuresis through renal tubular mechanisms, whereas SGLT2is cause osmotic diuresis through urinary glucose excretion. When used together, this dual mechanism might lead to excessive fluid loss in some patients, particularly those with pre‐existing heart disease who may be less able to compensate for volume changes. The finding that cardiovascular harm was concentrated specifically in patients with established cardiovascular disease, whereas those without prior disease experienced cardiovascular benefit, suggests that patients with more advanced heart disease may be particularly vulnerable to these potential interactions.

The balance between mortality and cardiovascular outcomes has significant clinical consequences. Although combination therapy offers survival benefits, it also leads to an increase in cardiovascular incidents, particularly among patients with pre‐existing cardiovascular conditions, who are frequently the focus of aggressive cardiometabolic risk management. This contradictory result calls for personalized treatment plans that consider conflicting outcome priorities rather than automatically opting for combination therapy methods. For patients prioritizing cardiovascular event prevention or those with a history of myocardial infarction, stroke, or heart failure, these data suggest GLP‐1 RA monotherapy may be preferred, pending confirmatory randomized evidence. Conversely, for patients prioritizing mortality reduction without established cardiovascular disease, combination therapy may be associated with favorable mortality profiles, though the early cardiovascular signal and residual confounding preclude a firm recommendation.

Our hepatic findings corroborate existing mechanistic and observational evidence while clarifying the magnitude of the benefits achievable with combination therapy. High‐quality randomized trials have established hepatoprotective effects for both agent classes: Semaglutide demonstrated substantial histologic resolution of steatohepatitis without worsening fibrosis compared with placebo in the ESSENCE trial [31], whereas dapagliflozin achieved significant MASH improvement rates [14]. Network meta‐analyses comparing these agents have shown that GLP‐1 RAs, particularly semaglutide and liraglutide, rank highest for weight‐mediated hepatic fat reduction and overall histological improvement, whereas SGLT2i demonstrate strong effects on enzymatic normalization and fibrosis markers through complementary metabolic mechanisms [11, 13, 32].

However, the lack of significant hepatic advantage of combination therapy versus GLP‐1 RA monotherapy suggests potential maximum effects for some hepatic endpoints in this population. A prior TriNetX analysis reported substantial reductions in major liver outcomes with GLP‐1 RA versus SGLT2i [5], whereas our bias‐corrected combination therapy estimates showed minimal benefit versus SGLT2i, falling between the two monotherapy options rather than substantially exceeding both. This pattern indicates that GLP‐1 RA monotherapy may already approach near‐maximal efficacy for preventing clinical hepatic decompensation events in MASLD + T2DM, with SGLT2i addition providing a modest incremental benefit but not the dramatic synergy one might expect from combining agents with complementary hepatoprotective mechanisms. Alternative explanations include residual confounding by hepatic fibrosis severity, as FIB‐4, albumin, and bilirubin were unavailable for PSM, and follow‐up duration insufficient to capture histological progression events in this population. The clinical implication is that hepatic outcome optimization alone does not justify combination therapy in most patients with MASLD + T2DM, given that GLP‐1 RA monotherapy achieves comparable hepatoprotection without the cardiovascular harm signal observed with combination therapy.

Our BHBE correction framework revealed that substantial proportions of the observed treatment effects were attributable to unmeasured confounding, with bias correction reversing the direction of cardiovascular associations from apparent benefit to harm. This finding has critical methodological implications: Even with rigorous PSM across measured baseline covariates, on which HbA1c, LVEF, and BMI remained imbalanced, residual confounding substantially influences the effect estimates in observational pharmacoepidemiology. Our transparent quantification of bias magnitude and demonstration that conclusions can reverse direction after appropriate adjustment provides an important calibration for interpreting observational comparative effectiveness studies and underscores the necessity of sensitivity analyses and bias correction methods beyond traditional matching or weighting approaches [15, 22].

The main strengths of this study are as follows: It had a large sample size from a variety of healthcare organizations across the United States; it had a three pairwise emulation design that allowed for simultaneous evaluation of combination therapy versus both monotherapy strategies; it had rigorous PSM that achieved balance on most measured covariates, with residual imbalance in HbA1c, LVEF, and BMI; it used BHBE correction with empirically calibrated priors to quantify unmeasured confounding; it had follow‐up of up to 5 years (median 1.7–2.2 years across comparisons); it had a comprehensive evaluation across mortality, cardiovascular, and hepatic outcome domains; it had prespecified subgroup analyses that identified critical effect modification by baseline cardiovascular disease status; and it adhered to TARGET reporting guidelines for target trial emulation studies [16, 17, 18, 19, 20, 21, 22].

Several limitations warrant consideration when interpreting the findings of this study. First, even with strict control of confounding variables, it is impossible to completely eliminate residual unmeasured confounding inherent to observational designs. This is especially true for disease severity markers that are not included in administrative data, frailty indices, or prescription patterns. This implies that effect estimates may still be somewhat biased, even with methodological safeguards. Second, outcomes were established using diagnostic codes without independent validation, which may have introduced potential misclassifications. Although this study utilized validated code sets, the absence of clinical validation means that some events may represent misclassified conditions, and hepatic outcomes lack histologic confirmation, limiting the certainty that the observed associations reflect actual clinical events. Third, this study could not assess treatment adherence, discontinuation, or switching patterns, which may have biased effect estimates in either direction; differential discontinuation due to adverse events in the combination arm could inflate apparent benefits, whereas discontinuation in the comparator arm could attenuate them. Structural sensitivity analyses evaluating alternative combination definition windows and treatment‐switching definitions were not performed and represent a priority for future replication studies.

Fourth, the absence of liver histology precludes the evaluation of fibrosis progression, with our composite outcome capturing late‐stage decompensation rather than earlier histologic improvements, limiting its applicability to patients seeking to prevent fibrosis advancement. The observed attenuation of hepatic benefit in patients with NASH compared with NAFLD‐only patients may reflect more advanced baseline fibrosis in the NASH subgroup, where clinical endpoints are less responsive to pharmacological intervention over the follow‐up duration studied. Fifth, the data source, although geographically diverse, predominantly represents academic medical centers, potentially limiting generalizability to community practice or international populations, meaning associations may not hold in systems with different prescribing practices. Additionally, as a proprietary federated platform, TriNetX cohort construction, PSM, and covariate balance outputs cannot be independently reproduced outside the platform environment, limiting external verifiability of these analytical steps. A substantial proportion of GLP‐1 RA monotherapy initiators were excluded by the caliper restriction in the GLP‐1 RA versus SGLT2i comparison, retaining only the most clinically similar patients; findings from this comparison may therefore not generalize to the broader GLP‐1 RA monotherapy population.

Sixth, laboratory values demonstrated notable missingness, necessitating a hybrid missing‐data approach (exclusion for variables > 20% missing, missingness indicators for the remainder) that may introduce selection bias if missingness correlates with disease severity, potentially overestimating benefits if sicker patients were systematically excluded. Additionally, although this study implemented landmark analysis to minimize immortal time bias, patients initiating combination therapy inherently survived longer to receive both medications, and residual bias may persist, potentially partially explaining mortality benefits if combination initiators represent healthier phenotypes. Finally, reverse Kaplan–Meier analyses demonstrated consistently higher observation probabilities in the treatment arm across all three comparisons, suggesting nonrandom administrative censoring favoring the treatment arm; this informative censoring pattern may have introduced bias in the direction of overestimating treatment benefits.

Our findings have identified several critical priorities. First, RCTs directly comparing combination therapy versus GLP‐1 RA monotherapy with adequately powered cardiovascular endpoints are urgently needed to definitively establish whether the MACE harm signal represents a causal risk or residual confounding from unmeasured patient selection factors. Second, mechanistic studies utilizing detailed hemodynamic monitoring, cardiac imaging, and metabolic profiling should investigate potential pharmacodynamic interactions between GLP‐1 RAs and SGLT2i that might explain the increased cardiovascular events in patients with established disease, including the assessment of volume status, cardiac output, and renal perfusion patterns. Third, trials should evaluate the optimal combination therapy duration before stepping down to monotherapy. Since the cardiovascular signal in our analysis was concentrated in the first year of treatment and did not persist thereafter, trials examining whether early monitoring and step‐down to GLP‐1 RA monotherapy after the high‐risk early period might preserve mortality benefit while minimizing cardiovascular risk would be informative.

Fourth, studies incorporating serial liver histology or advanced imaging endpoints (MRI‐PDFF, magnetic resonance elastography) would clarify whether combination therapy provides histological benefits beyond the clinical outcome advantages observed in this study and whether true ceiling effects exist for hepatoprotection in the long term. Fifth, cost‐effectiveness studies should formally assess whether the added expense of dual therapy is justified, given that it reduces mortality but increases cardiovascular events, particularly to guide resource allocation decisions and help clinicians and patients weigh competing outcome priorities. Sixth, the investigation of effect modification by specific cardiovascular disease subtypes, including heart failure with preserved versus reduced ejection fraction, ischemic versus nonischemic cardiomyopathy, and coronary versus cerebrovascular disease, may identify subpopulations in which the cardiovascular safety profile of the combination therapy differs significantly. Finally, external validation in independent cohorts, including non‐US healthcare systems, Asian populations where MASLD prevalence and cardiovascular risk profiles differ, and real‐world community practice registries, would establish the generalizability and reproducibility of our findings across diverse clinical contexts and healthcare delivery systems. External validation of the BHBE correction framework against comparisons with known effect sizes from randomized trials represents an important methodological priority arising from this work.

## 5 Conclusion

In patients with MASLD and T2DM, combination GLP‐1 RA plus SGLT2i therapy was associated with mortality reduction but increased cardiovascular events compared with GLP‐1 RA monotherapy, a signal concentrated in the first year of treatment, not sustained in the landmark analysis, and most pronounced in patients with established cardiovascular disease. After bias correction, the apparent cardiovascular trend favoring combination over SGLT2i monotherapy was reversed and attributed to residual confounding, whereas the mortality advantage persisted. Hepatic benefits were modest and did not exceed those of GLP‐1 RA monotherapy. These hypothesis‐generating findings suggest that combination therapy may be preferred for mortality reduction in patients without prior cardiovascular disease, whereas GLP‐1 RA monotherapy may be preferred when cardiovascular event prevention is the primary goal, pending randomized confirmation.

## Funding

The authors have nothing to report.

## Ethics Statement

This study utilized deidentified data from the TriNetX Research Network in accordance with the Declaration of Helsinki. All data are deidentified in compliance with HIPAA Privacy Rule §164.514(a); this analysis did not constitute human‐subjects research, and institutional review board approval and informed consent were not required. TriNetX maintains institutional review board compliance and conducts regular data quality assessments to validate data completeness and accuracy.

## Conflicts of Interest

The authors declare no conflicts of interest.

## Supporting information


**Table S1:** TARGET checklist for transparent reporting of target trial emulation studies.
**Table S2:** Target trial specification and emulation protocol.
**Table S3:** Variable definitions, code lists, and operationalization.
**Table S4:** Participant flow and cohort attrition.
**Table S5:** Propensity score model specification, covariate balance, and matching diagnostics for three pairwise target trial emulation comparisons.
**Table S6:** Bayesian hierarchical bias‐effect model specification, MCMC diagnostics, and posterior summary.
**Table S7:** Subgroup analyses by baseline characteristics for primary comparison (combination versus SGLT2i) (PSM‐adjusted HRs; for BHBE‐corrected subgroup estimates see Figure 6).
**Table S8:** Missing data assessment by treatment group.
**Figure S1:** Propensity score distribution before and after matching.
**Figure S2:** Cumulative incidence curves for all comparisons.
**Figure S3:** Time‐varying hazard ratios across follow‐up periods.
**Figure S4:** Reverse Kaplan–Meier curves for follow‐up completeness.
**Figure S5:** Bayesian hierarchical bias‐effect model computational framework.

## Data Availability

The data that support the findings of this study are available from the TriNetX Research Network, but restrictions apply to the availability of these data, which were accessed under institutional agreement and are not publicly available. Data are available from TriNetX for researchers who meet the criteria for access to confidential data through institutional collaboration agreements with participating healthcare organizations.
