# Supplementary material for: GLP‐1 RA Plus SGLT2i Versus Monotherapy in MASLD and Type 2 Diabetes: Three Pairwise Target Trial Emulations
Source: J Gastroenterol Hepatol. 2026 Jul 13;41(9):2862–81. doi: 10.1111/jgh.70589 (PMC13534337; doi:10.1111/jgh.70589)
Supplement: Supplementary file 1 [file JGH-41-2862-s001.pdf]

Supplementary Material

GLP-1 RA Plus SGLT2i Versus Monotherapy in MASLD and Type 2 Diabetes: Three Pairwise Target Trial Emulations

The authors have provided this supplementary material to demonstrate additional information about this study.

Legends:

Table S1: TARGET Checklist for Transparent Reporting of Target Trial Emulation Studies.

Table S2: Target Trial Specification and Emulation Protocol.

Table S3: Variable Definitions, Code Lists, and Operationalization

Table S4: Participant Flow and Cohort Attrition.

Table S5: Propensity Score Model Specification, Covariate Balance, and Matching Diagnostics for Three Pairwise Target Trial Emulation Comparisons

Table S6: Bayesian Hierarchical Bias-Effect Model Specification, MCMC Diagnostics, and Posterior Summary

Table S7: Subgroup Analyses by Baseline Characteristics for Primary Comparison (Combination vs SGLT2i) (PSM-adjusted HRs; for BHBE-corrected subgroup estimates see Figure 6)

Table S8: Missing Data Assessment by Treatment Group.

Figure S1: Propensity Score Distribution Before and After Matching.

Figure S2: Cumulative Incidence Curves for All Comparisons.

Figure S3: Time-Varying Hazard Ratios Across Follow-Up Periods.

Figure S4: Reverse Kaplan-Meier Curves for Follow-Up Completeness.

Figure S5: Bayesian Hierarchical Bias-Effect Model Computational Framework.

Table S1: TARGET Checklist for Transparent Reporting of Target Trial Emulation Studies.

Item	Checklist Element	Description	Reported	Manuscript Location
**Title and Abstract:**				
1	Title identification	Identify the study as a target trial emulation in the title	Yes	Title
2	Abstract summary	Provide a structured summary including target trial specification, data source, and key findings	Yes	Abstract
**Introduction:**				
3	Background rationale	Explain the scientific background and rationale for emulating a target trial	Yes	Introduction, ¶1–2
4	Objectives	State specific objectives, including the target trial being emulated	Yes	Introduction, ¶3
**Methods — Target Trial Specification:**				
5	Eligibility criteria	Specify eligibility criteria for the target trial	Yes	Methods, "Target Trial Specification"; Table S3
6	Treatment strategies	Define the treatment strategies being compared in the target trial	Yes	Methods, "Treatment Strategies"; Table S3
7	Treatment assignment	Describe how participants would be assigned to treatment in the target trial	Yes	Methods, "Assignment Procedures"; Table S3
8	Outcome	Define the primary and secondary outcomes for the target trial	Yes	Methods 2.5; Table S3
9	Follow-up	Specify the start and end of follow-up for the target trial	Yes	Methods, "Follow-up Period"
10	Causal contrast	State the causal contrast of interest (intention-to-treat, per-protocol, or other)	Yes	Methods, "Statistical Analysis"
**Methods — Emulation:**				
11	Data source	Describe the data source(s) used for emulation, including setting and dates of data collection	Yes	Methods, "Data Source"
12	Eligibility emulation	Describe how target trial eligibility criteria were emulated using the data source	Yes	Methods, "Cohort Selection"; Table S1
13	Treatment emulation	Describe how treatment strategies were emulated, including definitions and coding	Yes	Methods, "Exposure Definition"; Table S1
14	Assignment emulation	Describe how treatment assignment was emulated (e.g., time zero, grace period)	Yes	Methods, "Index Date Definition"
15	Outcome emulation	Describe how outcomes were identified and validated in the data source	Yes	Methods, "Outcome Ascertainment"; Table S1
16	Follow-up emulation	Describe how follow-up was implemented, including censoring	Yes	Methods, "Follow-up and Censoring"
17	Confounding adjustment	Describe methods used to control for confounding (e.g., PSM, weighting, regression)	Yes	Methods, "Propensity Score Matching"; Table S5
18	Sensitivity analyses	Describe any sensitivity analyses planned to assess robustness	Yes	Methods, "Sensitivity Analyses"; Table 4
**Results:**				
19	Participants	Report numbers of individuals at each stage of study inclusion with flow diagram	Yes	Results, "Study Population"; Table S4; Figure 1
20	Main results	Report main results with measures of precision (e.g., 95% CI)	Yes	Results; Tables 2–3
21	Sensitivity results	Report results of sensitivity analyses	Yes	Results, "Sensitivity Analyses"; Table 4
**Discussion:**				
22	Key findings	Summarize key findings with reference to study objectives	Yes	Discussion, ¶1
23	Limitations	Discuss limitations of the emulation approach and data source	Yes	Discussion, "Limitations"
24	Interpretation	Provide cautious interpretation considering emulation-specific issues	Yes	Discussion, "Clinical Implications"
25	Generalizability	Discuss generalizability of findings to the target trial population	Yes	Discussion, "Generalizability"
**Other Information:**				
26	Funding	Describe sources of funding and role of funders	Yes	Funding Statement
27	Data access	Describe data access and verification procedures	Yes	Data Availability Statement
28	Protocol registration	State whether study protocol was registered and where	No — this observational study was not prospectively registered	Methods, ¶1

Abbreviations: *CI, confidence interval; PSM, propensity score matching; TARGET, TArgeted trial emulation Good practices for Emulated Trials.* ***Checklist Reference:*** *This checklist is adapted from the TARGET guidance for reporting target trial emulation studies (Cashin et al. Development of the TArgeted trial emulation Good practices for Emulated Trials [TARGET] reporting guideline. JAMA 2025). The TARGET framework provides standardized reporting requirements to enhance transparency and reproducibility of target trial emulation studies using real-world data.* ***Compliance Summary:*** *This study adheres to all 28 items of the TARGET checklist. Key methodological elements include: (1) explicit specification of the target trial protocol (Table S3), (2) transparent reporting of emulation decisions and their justifications, (3) comprehensive description of confounding adjustment via propensity score matching with covariate balance assessment, (4) pre-specified sensitivity analyses including Bayesian bias correction (BHBE model), and (5) clear acknowledgment of emulation-specific limitations including unmeasured confounding and informative censoring.* ***Deviations from Ideal Target Trial:*** *The principal deviations from an ideal randomized trial include: (1) non-random treatment assignment addressed via propensity score matching, (2) potential for unmeasured confounding addressed via BHBE bias correction framework, (3) informative censoring addressed via sensitivity analysis, and (4) outcome ascertainment based on diagnostic codes rather than adjudicated events.*

Table S2: Target Trial Specification and Emulation Protocol.

Protocol Component	Target Trial Specification	Emulation Using TriNetX Data
**Eligibility Criteria:**		
Age	Adults aged ≥18 years	Adults aged ≥18 years at index date
Diagnosis	Confirmed MASLD/NASH with concurrent type 2 diabetes mellitus	≥1 ICD-10-CM diagnosis code for NAFLD (K76.0) or NASH (K75.81) AND ≥1 code for T2DM (E11.x) any time before index
Liver disease exclusions	No hemochromatosis, alcoholic liver disease, autoimmune hepatitis, or viral hepatitis	No ICD-10-CM codes for E83.11 (hemochromatosis), K70.x (alcoholic liver disease), K75.4 (autoimmune hepatitis), or B15–B19 (viral hepatitis) any time in record
Treatment history	Treatment-naïve to study medications or initiating new therapy	First prescription of qualifying medication(s) following MASLD+T2DM diagnosis
**Treatment Strategies:**		
Intervention arm 1	Initiate GLP-1 RA + SGLT2i combination therapy	First overlapping prescriptions of ATC class A10BJ (GLP-1 RA) AND A10BK (SGLT2i) after MASLD+T2DM diagnosis
Comparator arm 2	Initiate GLP-1 RA monotherapy (no SGLT2i)	First prescription of A10BJ without any concurrent or subsequent A10BK prescription
Comparator arm 3	Initiate SGLT2i monotherapy (no GLP-1 RA)	First prescription of A10BK without any concurrent or subsequent A10BJ prescription
**Treatment Assignment:**		
Randomization	Random assignment to treatment strategy at baseline	No randomization; observational treatment assignment
Allocation concealment	Concealed allocation	Not applicable
Blinding	Double-blind (participants and investigators)	Open-label; no blinding
Confounding control	Randomization eliminates confounding	Propensity score 1:1 nearest-neighbor matching (caliper 0.1) on 47 baseline covariates; residual confounding addressed via BHBE bias correction
**Causal Contrast:**		
Primary estimand	Intention-to-treat effect	Intention-to-treat analogue: patients analyzed according to initial treatment assignment regardless of subsequent treatment changes
Per-protocol	Per-protocol effect with adherence monitoring	Not estimated; treatment discontinuation/switching not captured
**Time Zero (Index Date):**		
Definition	Date of randomization	Date of first qualifying medication prescription following MASLD+T2DM diagnosis
Grace period	Same-day assignment	Combination therapy: both medications initiated within 30-day window; monotherapy: single medication with no comparator class within entire follow-up
**Outcomes:**		
Primary outcome 1	All-cause mortality	Death flag in TriNetX mortality data; ascertained via Social Security Death Index linkage and institutional records
Primary outcome 2	Major adverse cardiovascular events (MACE): composite of acute MI, stroke, heart failure hospitalization, cardiac arrest	First occurrence of ICD-10-CM codes: I21.x (MI), I60, I61, I62, I63, I63.50 (stroke), I50.x (heart failure), I46.x (cardiac arrest)
Primary outcome 3	Composite liver outcomes: cirrhosis, hepatic fibrosis, ascites, hepatic encephalopathy, hepatic failure, HCC	First occurrence of ICD-10-CM codes: K74.x (cirrhosis/fibrosis), R18.x (ascites), K76.82 (encephalopathy), K72.x (hepatic failure), C22.0/ICD-O-3 8170/3 (HCC)
Secondary outcome	Hepatocellular carcinoma (individual endpoint)	First occurrence of C22.0 or ICD-O-3 morphology code 8170/3
Outcome adjudication	Independent blinded adjudication committee	Algorithm-based ascertainment using validated ICD-10-CM codes; no clinical adjudication; algorithm-based ICD code ascertainment; no independent adjudication committee
**Follow-up Period:**		
Start of follow-up	Day of randomization (time zero)	Day 1 after index date (1-day landmark to avoid immortal time bias)
End of follow-up	5 years after randomization, outcome occurrence, death, or loss to follow-up	1,825 days (5 years) after index, outcome occurrence, death, or last recorded healthcare encounter (administrative censoring)
Minimum follow-up	12 months	No minimum required; all eligible patients included
**Analysis Plan:**		
Primary analysis	Cox proportional hazards regression	Cox proportional hazards regression with robust variance estimation
Effect measure	Hazard ratio with 95% confidence interval	Hazard ratio with 95% CI; log-rank test for survival comparison
Proportional hazards	Assessed via Schoenfeld residuals	Assessed via Schoenfeld residuals; time-varying HRs estimated when PH violated
Subgroup analyses	Pre-specified by age, sex, baseline CVD, CKD stage, HbA1c, BMI	Pre-specified subgroup analyses (Table S8)
Sensitivity analyses	Multiple imputation for missing data; per-protocol analysis	BHBE Bayesian bias correction; prior sensitivity analysis (7 scenarios)
**Sample Size:**		
Power calculation	90% power to detect HR 0.80 for primary outcome at α=0.05	Observational study; no formal power calculation; effective sample size reported post-matching (post-hoc, calculated after cohort sizes were determined)
Planned enrollment	Estimated N=40,000 per arm	Combination: 42,518; GLP-1 RA: 84,562; SGLT2i: 60,682 (pre-matching)
**Data Collection:**		
Data source	Multi-center prospective trial with standardized case report forms	TriNetX federated research network: electronic health records from 110 US healthcare organizations
Data quality	Centralized data management with range checks and queries	Distributed data model with local quality checks; no centralized adjudication
Missing data	Minimized via protocol; multiple imputation for residual missingness	Hybrid approach: variables >20% missing excluded from PS model; variables ≤20% missing retained with binary missingness indicators

Abbreviations: *BHBE, Bayesian Hierarchical Bias-Effect model; BMI, body mass index; CI, confidence interval; CKD, chronic kidney disease; CVD, cardiovascular disease; GLP-1 RA, glucagon-like peptide-1 receptor agonist; HbA1c, glycated hemoglobin; HCC, hepatocellular carcinoma; HR, hazard ratio; ICD-10-CM, International Classification of Diseases 10th Revision Clinical Modification; ICD-O-3, International Classification of Diseases for Oncology 3rd Edition; MACE, major adverse cardiovascular events; MASLD, metabolic dysfunction-associated steatotic liver disease; MI, myocardial infarction; NAFLD, nonalcoholic fatty liver disease; NASH, nonalcoholic steatohepatitis; PH, proportional hazards; PS, propensity score; SGLT2i, sodium-glucose cotransporter-2 inhibitor; T2DM, type 2 diabetes mellitus.*

Table S3: Variable Definitions, Code Lists, and Operationalization

Category	Variable	Description	Code System	Code(s)	Operationalization
EXPOSURES					
Treatment	GLP-1 Receptor Agonists	Glucagon-like peptide-1 analogues	ATC	A10BJ	Any prescription record
	SGLT2 Inhibitors	Sodium-glucose cotransporter-2 inhibitors	ATC	A10BK	Any prescription record
	Combination Therapy	GLP-1 RA + SGLT2i concurrent use	ATC	A10BJ AND A10BK	Both medications with overlapping dates within 30-day window
ELIGIBILITY CRITERIA — INCLUSION					
Inclusion	NAFLD/NASH	Nonalcoholic steatohepatitis	ICD-10-CM	K75.81	≥1 diagnosis code any time before index
	Fatty Liver Disease	Fatty (change of) liver, NEC	ICD-10-CM	K76.0	≥1 diagnosis code any time before index
	Type 2 Diabetes Mellitus	Type 2 diabetes mellitus	ICD-10-CM	E11 (all subcodes)	≥1 diagnosis code any time before index
	Age	Adults ≥18 years	Demographics	—	Age at index date
ELIGIBILITY CRITERIA — EXCLUSION					
Exclusion	Hemochromatosis	Hereditary hemochromatosis	ICD-10-CM	E83.11	Any diagnosis code
	Alcoholic Liver Disease	Alcoholic liver disease	ICD-10-CM	K70 (all subcodes)	Any diagnosis code
	Autoimmune Hepatitis	Autoimmune hepatitis	ICD-10-CM	K75.4	Any diagnosis code
	Viral Hepatitis	Viral hepatitis B and C	ICD-10-CM	B15–B19	Any diagnosis code
PRIMARY OUTCOMES					
Outcome	All-Cause Mortality	Death from any cause	TriNetX	Death Flag	Standard mortality indicator
	MACE (Composite)	Major adverse cardiovascular events	ICD-10-CM	See components below	First occurrence of any component
	— Acute MI	Acute myocardial infarction (including subsequent MI)	ICD-10-CM	I21, I22	≥1 diagnosis code
	— Stroke	Ischaemic and haemorrhagic cerebrovascular events	ICD-10-CM	I60, I61, I62, I63, I63.50	≥1 diagnosis code
	— Heart Failure	Heart failure	ICD-10-CM	I50, I50.1–I50.9	≥1 diagnosis code
	— Cardiac Arrest	Cardiac arrest	ICD-10-CM	I46, I46.2, I46.8, I46.9	≥1 diagnosis code
	Composite Liver Outcome (Composite)	Liver-related adverse outcomes	ICD-10-CM/ICD-O-3	See components below	First occurrence of any component
	— Cirrhosis, unspecified	Unspecified cirrhosis of liver	ICD-10-CM	K74.60	≥1 diagnosis code
	— Cirrhosis, other	Other cirrhosis of liver	ICD-10-CM	K74.69	≥1 diagnosis code
	— Cirrhosis NOS	Other and unspecified cirrhosis	ICD-10-CM	K74.6	≥1 diagnosis code
	— Fibrosis and cirrhosis	Fibrosis and cirrhosis of liver	ICD-10-CM	K74	≥1 diagnosis code
	— Hepatic fibrosis	Hepatic fibrosis	ICD-10-CM	K74.0	≥1 diagnosis code
	— Fibrosis with sclerosis	Hepatic fibrosis with sclerosis	ICD-10-CM	K74.2	≥1 diagnosis code
	— Ascites	Ascites	ICD-10-CM	R18	≥1 diagnosis code
	— Ascites, other	Other ascites	ICD-10-CM	R18.8	≥1 diagnosis code
	— Hepatic failure	Hepatic failure, NEC	ICD-10-CM	K72 (all subcodes)	≥1 diagnosis code
	— Portal hypertension[a]	Chronic passive congestion of liver	ICD-10-CM	K76.1	≥1 diagnosis code
	— Hepatic encephalopathy	Hepatic encephalopathy	ICD-10-CM	K76.82	≥1 diagnosis code
	— HCC (morphology)	Hepatocellular carcinoma, NOS	ICD-O-3	8170/3	≥1 diagnosis code
	— HCC (topography)	Liver cell carcinoma	ICD-10-CM	C22.0	≥1 diagnosis code
SECONDARY OUTCOME					
Outcome	Hepatocellular Carcinoma	HCC as individual endpoint	ICD-10-CM/ICD-O-3	C22.0, 8170/3	≥1 diagnosis code
COVARIATES					
Demographic	Age	Age at index date	Demographics	—	Continuous, years
	Sex	Biological sex	Demographics	—	Male/Female
	Race	Self-reported race	Demographics	—	White/Black/Asian/Other
	Ethnicity	Hispanic/Latino ethnicity	Demographics	—	Hispanic/Non-Hispanic
Comorbidity	Hypertension	Essential hypertension	ICD-10-CM	I10	≥1 diagnosis code before index
	Dyslipidemia	Disorders of lipoprotein metabolism	ICD-10-CM	E78 (all subcodes)	≥1 diagnosis code before index
	Coronary Artery Disease	Ischemic heart disease	ICD-10-CM	I25 (all subcodes)	≥1 diagnosis code before index
	Chronic Kidney Disease	Chronic kidney disease	ICD-10-CM	N18 (all subcodes)	≥1 diagnosis code before index
	Obesity	Obesity	ICD-10-CM	E66 (all subcodes)	≥1 diagnosis code before index
Laboratory	HbA1c	Glycated haemoglobin	LOINC	4548-4, 17856-6	Most recent value before index, %
	eGFR	Estimated glomerular filtration rate	LOINC	33914-3, 48642-3, 48643-1	Most recent value before index, mL/min/1.73m²
	ALT	Alanine aminotransferase	LOINC	1742-6, 1743-4	Most recent value before index, U/L
	AST	Aspartate aminotransferase	LOINC	1920-8	Most recent value before index, U/L
Vital Signs	BMI	Body mass index	LOINC	39156-5	Most recent value before index, kg/m²
	Systolic BP	Systolic blood pressure	LOINC	8480-6	Most recent value before index, mmHg
	Diastolic BP	Diastolic blood pressure	LOINC	8462-4	Most recent value before index, mmHg

[a] Code K76.1 (chronic passive congestion of liver) may capture hepatic congestion secondary to right heart failure in patients with cardiovascular comorbidities, and is not exclusive to hepatic aetiology. This represents a potential source of outcome misclassification within the composite liver endpoint.

Abbreviations: ATC, Anatomical Therapeutic Chemical; BMI, body mass index; BP, blood pressure; Combo, GLP-1 RA + SGLT2i combination therapy; eGFR, estimated glomerular filtration rate; GLP-1 RA, glucagon-like peptide-1 receptor agonist; HbA1c, glycated haemoglobin; HCC, hepatocellular carcinoma; ICD-10-CM, International Classification of Diseases, 10th Revision, Clinical Modification; ICD-O-3, International Classification of Diseases for Oncology, 3rd Edition; LOINC, Logical Observation Identifiers Names and Codes; MACE, major adverse cardiovascular events; NEC, not elsewhere classified; NOS, not otherwise specified; SGLT2i, sodium-glucose cotransporter-2 inhibitor.

Table S4: Participant Flow and Cohort Attrition.

Stage	Description	Combination Therapy	GLP-1 RA Monotherapy	SGLT2i Monotherapy	Exclusion Reason
**IDENTIFICATION:**					
1	TriNetX Research Network total patients	—	—	—	Source population
2	Patients with NAFLD or NASH diagnosis (K75.81, K76.0)	892,456	892,456	892,456	—
3	Patients with concurrent T2DM diagnosis (E11.x)	487,329	487,329	487,329	No T2DM: 405,127
**ELIGIBILITY SCREENING:**					
4	Adults aged ≥18 years	486,847	486,847	486,847	Age <18: 482
	Age <18 years excluded: n=482	—	—	—	Age <18
5	Excluded: Hemochromatosis (E83.11)	485,192	485,192	485,192	Hemochromatosis: 1,655
6	Excluded: Alcoholic liver disease (K70.x)	461,438	461,438	461,438	Alcoholic liver disease: 23,754
7	Excluded: Autoimmune hepatitis (K75.4)	458,726	458,726	458,726	Autoimmune hepatitis: 2,712
8	Excluded: Viral hepatitis (B15–B19)	438,291	438,291	438,291	Viral hepatitis: 20,435
9	Eligible MASLD + T2DM population	438,291	438,291	438,291	—
**TREATMENT CLASSIFICATION:**					
10	Received GLP-1 RA (A10BJ) any time after diagnosis	127,244	127,244	—	—
11	Received SGLT2i (A10BK) any time after diagnosis	94,344	—	94,344	—
12	Received both GLP-1 RA + SGLT2i (combination)	42,518	Excluded	Excluded	—
13	Received GLP-1 RA only (no SGLT2i ever)	Excluded	84,562	Excluded	—
14	Received SGLT2i only (no GLP-1 RA ever)	Excluded	Excluded	60,682	—
**PRE-MATCHING COHORTS:**					
15	Analytic cohort before PSM	42,518	84,562	60,682	—
**PROPENSITY SCORE MATCHING:**					
	Comparison 1: Combination vs GLP-1 RA				
16a	Combination cohort for matching	42,518	—	—	—
16b	GLP-1 RA cohort for matching	—	84,562	—	—
17a	Matched combination patients	42,423	—	—	Unmatched: 95
17b	Matched GLP-1 RA patients	—	42,423	—	Unmatched: 42,303
	Comparison 2: Combination vs SGLT2i				
18a	Combination cohort for matching	42,518	—	—	—
18b	SGLT2i cohort for matching	—	—	60,682	—
19a	Matched combination patients	40,349	—	—	Unmatched: 2,169
19b	Matched SGLT2i patients	—	—	40,349	Unmatched: 11,477
	Comparison 3: GLP-1 RA vs SGLT2i				
20a	GLP-1 RA cohort for matching	—	84,562	—	—
20b	SGLT2i cohort for matching	—	—	60,682	—
21a	Matched GLP-1 RA patients	—	60,682	—	Unmatched: 32,736
21b	Matched SGLT2i patients	—	—	60,682	Unmatched: 8,856
**OUTCOME-SPECIFIC EXCLUSIONS:**					
	All-Cause Mortality Analysis				
22	Combo vs GLP-1 RA: Excluded for prior death	159	102	—	Death before index
23	Combo vs GLP-1 RA: Final mortality cohort	42,264	42,321	—	—
24	Combo vs SGLT2i: Excluded for prior death	154	—	89	Death before index
25	Combo vs SGLT2i: Final mortality cohort	40,195	—	40,260	—
26	GLP-1 RA vs SGLT2i: Excluded for prior death	—	111	118	Death before index
27	GLP-1 RA vs SGLT2i: Final mortality cohort	—	51,715	51,708	—
	MACE Analysis				
28	Combo vs GLP-1 RA: Excluded for prior MACE[a]	11,700	8,735	—	MACE before index
29	Combo vs GLP-1 RA: Final MACE cohort	30,723	33,688	—	—
30	Combo vs SGLT2i: Excluded for prior MACE	11,397	—	13,869	MACE before index
31	Combo vs SGLT2i: Final MACE cohort	28,952	—	26,480	—
32	GLP-1 RA vs SGLT2i: Excluded for prior MACE	—	10,798	17,918	MACE before index
33	GLP-1 RA vs SGLT2i: Final MACE cohort	—	41,028	33,908	—
	Composite Liver Outcomes Analysis				
34	Combo vs GLP-1 RA: Excluded for prior liver outcome	5,913	5,505	—	Liver outcome before index
35	Combo vs GLP-1 RA: Final liver cohort	36,510	36,918	—	—
36	Combo vs SGLT2i: Excluded for prior liver outcome	5,685	—	6,082	Liver outcome before index
37	Combo vs SGLT2i: Final liver cohort	34,664	—	34,267	—

38	GLP-1 RA vs SGLT2i: Excluded for prior liver outcome	—	6,945	7,928	Liver outcome before index
39	GLP-1 RA vs SGLT2i: Final liver cohort	—	44,881	43,898	—
	Hepatocellular Carcinoma Analysis				
40	Combo vs GLP-1 RA: Excluded for prior HCC	231	227	—	HCC before index
41	Combo vs GLP-1 RA: Final HCC cohort	42,192	42,196	—	—
42	Combo vs SGLT2i: Excluded for prior HCC	226	—	244	HCC before index
43	Combo vs SGLT2i: Final HCC cohort	40,123	—	40,105	—
44	GLP-1 RA vs SGLT2i: Excluded for prior HCC	—	313	338	HCC before index
45	GLP-1 RA vs SGLT2i: Final HCC cohort	—	51,513	51,488	—

Abbreviations: *GLP-1 RA, glucagon-like peptide-1 receptor agonist; HCC, hepatocellular carcinoma; MACE, major adverse cardiovascular events; MASLD, metabolic dysfunction-associated steatotic liver disease; NAFLD, nonalcoholic fatty liver disease; NASH, nonalcoholic steatohepatitis; PSM, propensity score matching; SGLT2i, sodium-glucose cotransporter-2 inhibitor; T2DM, type 2 diabetes mellitus.* ***Data Source:*** *TriNetX Research Network, a federated health research network comprising 110 healthcare organizations across the United States, accessed January 2026.* ***Propensity Score Matching Specifications:*** *1:1 nearest-neighbor matching without replacement using a caliper width of 0.1 standard deviations of the logit of the propensity score. Matching performed separately for each pairwise comparison. Covariates included demographics (age, sex, race, ethnicity), comorbidities (hypertension, dyslipidemia, CAD, CKD, obesity, heart failure), laboratory values (HbA1c, eGFR, ALT, AST), and vital signs (BMI, systolic BP, diastolic BP).* ***Outcome-Specific Exclusions:*** *Patients with prevalent outcomes (occurring before the follow-up window start) were excluded from the respective outcome analysis to ensure incident outcome ascertainment. This approach prevents reverse causation and ensures appropriate risk set composition for survival analysis.*

[a] *Asymmetric prevalent-MACE exclusions for Comparison 1: 11,700/42,423 (27.6%) Combination vs 8,735/42,423 (20.6%) GLP-1 RA patients excluded for prior MACE.*

Table S5: Propensity Score Model Specification, Covariate Balance, and Matching Diagnostics for Three Pairwise Target Trial Emulation Comparisons

Characteristic	Combo Therapy	GLP-1 RA Mono	SMD	Combo Therapy	SGLT2i Mono	SMD	GLP-1 RA Mono	SGLT2i Mono	SMD
	Comparison 1: Combo vs GLP-1 RA			*Comparison 2: Combo vs SGLT2i*			*Comparison 3: GLP-1 RA vs SGLT2i*		
Model Specification and Diagnostics									
Matching method	1:1 nearest-neighbour, without replacement			1:1 nearest-neighbour, without replacement			1:1 nearest-neighbour, without replacement		
Caliper width	0.1 SD of logit PS			0.1 SD of logit PS			0.1 SD of logit PS		
Covariates in PS model, n	47			47			47		
PS estimation method	Logistic regression			Logistic regression			Logistic regression		
C-statistic (95% CI)	0.724 (0.719–0.729)			0.756 (0.751–0.761)			0.742 (0.738–0.746)		
Sample Sizes and Matching Rates									
Pre-match N (treatment arm)	42,518	—	—	42,518	—	—	60,682	—	—
Pre-match N (comparator arm)	—	84,562	—	—	60,682	—	—	60,682	—
Post-match pairs, n	42,423	42,423	—	40,349	40,349	—	60,682	60,682	—
Matching rate, treatment arm (%)	100.0	—	—	95.1	—	—	61.3	—	—
Matching rate, comparator arm (%)	—	50.2	—	—	66.5	—	—	85.4	—
Unmatched, treatment arm (n)	95	—	—	2,169	—	—	32,736	—	—
Unmatched, comparator arm (n)	—	42,139	—	—	20,333	—	—	8,856	—
Covariate Balance Summary									
Covariates assessed (TriNetX output), n	24			24			24		
Covariates with SMD >0.1 before matching, n	13			11			12		
Covariates with SMD >0.1 after matching, n	**4**			**3**			**5**		
Maximum SMD before matching	0.303			0.288			0.475		
Maximum SMD after matching	**0.303**			**0.223**			**0.426**		
Mean absolute SMD before matching	0.120			0.111			0.166		
Mean absolute SMD after matching	0.060			0.038			0.063		
Covariate Balance Before Propensity Score Matching									
Demographics									
Age, current (years), mean ± SD	61.5 ± 12.1	59.0 ± 13.3	0.196	61.5 ± 12.1	64.9 ± 12.6	0.276	59.0 ± 13.3	64.9 ± 12.6	0.456
Age at index (years), mean ± SD	58.3 ± 12.2	55.8 ± 13.2	0.202	58.3 ± 12.2	61.9 ± 12.8	0.288	55.8 ± 13.2	61.9 ± 12.8	0.475
Female sex (%)	55.5	62.5	0.144	55.5	48.4	0.142	62.5	48.4	0.287
White race (%)	71.3	71.9	0.014	71.3	68.2	0.068	71.9	68.2	0.081
Hispanic/Latino ethnicity (%)	13.5	13.0	0.015	13.5	11.6	0.056	13.0	11.6	0.041
Black/African American (%)	11.9	12.2	0.007	11.9	12.3	0.012	12.2	12.3	0.005
Diagnosis categories (ICD-10 chapter level)									
Endocrine/metabolic diseases, E00–E89 (%)	99.2	98.2	0.091	99.2	98.0	0.100	98.2	98.0	0.010
Digestive diseases, K00–K95 (%)	99.1	97.5	0.128	99.1	97.6	0.123	97.5	97.6	0.005
Circulatory diseases, I00–I99 (%)	91.7	85.9	0.185	91.7	91.2	0.020	85.9	91.2	0.165
Skin diseases, L00–L99 (%)	65.7	61.6	0.086	65.7	57.1	0.177	61.6	57.1	0.090
Blood/haematologic diseases, D50–D89 (%)	49.2	44.6	0.092	49.2	51.2	0.041	44.6	51.2	0.133
Medication categories									
Cardiovascular medications (%)	95.6	91.9	0.157	95.6	93.7	0.088	91.9	93.7	0.070
Other medications (%)	78.1	73.4	0.110	78.1	77.7	0.010	73.4	77.7	0.099
Laboratory values and vital signs									
HbA1c (%), mean ± SD	8.2 ± 1.8	7.6 ± 1.9	0.303	8.2 ± 1.8	7.8 ± 1.8	0.213	7.6 ± 1.9	7.8 ± 1.8	0.093
BMI (kg/m²), mean ± SD	36.1 ± 7.8	37.7 ± 8.0	0.197	36.1 ± 7.8	34.0 ± 7.8	0.281	37.7 ± 8.0	34.0 ± 7.8	0.474
LVEF (%), mean ± SD	57.8 ± 11.9	60.2 ± 9.9	0.217	57.8 ± 11.9	54.3 ± 14.5	0.266	60.2 ± 9.9	54.3 ± 14.5	0.475
LDL cholesterol (mg/dL), mean ± SD	85.3 ± 37.8	93.4 ± 37.8	0.212	85.3 ± 37.8	84.1 ± 38.0	0.033	93.4 ± 37.8	84.1 ± 38.0	0.245
Total cholesterol (mg/dL), mean ± SD	165.0 ± 48.6	172.9 ± 47.3	0.166	165.0 ± 48.6	162.0 ± 51.4	0.061	172.9 ± 47.3	162.0 ± 51.4	0.222
HDL cholesterol (mg/dL), mean ± SD	40.6 ± 14.3	41.9 ± 15.3	0.089	40.6 ± 14.3	40.3 ± 15.8	0.018	41.9 ± 15.3	40.3 ± 15.8	0.102
Triglycerides (mg/dL), mean ± SD	211.8 ± 216.1	198.0 ± 189.6	0.068	211.8 ± 216.1	198.4 ± 220.0	0.061	198.0 ± 189.6	198.4 ± 220.0	0.002
C-reactive protein (mg/L), mean ± SD	23.2 ± 45.8	21.6 ± 43.2	0.035	23.2 ± 45.8	28.4 ± 52.9	0.104	21.6 ± 43.2	28.4 ± 52.9	0.139
Heart rate (/min), mean ± SD	81.6 ± 14.5	81.3 ± 14.4	0.021	81.6 ± 14.5	80.0 ± 15.3	0.109	81.3 ± 14.4	80.0 ± 15.3	0.089
Systolic BP (mmHg), mean ± SD	130.0 ± 17.5	130.8 ± 17.2	0.045	130.0 ± 17.5	130.4 ± 19.2	0.019	130.8 ± 17.2	130.4 ± 19.2	0.023
Diastolic BP (mmHg), mean ± SD	76.2 ± 11.2	77.3 ± 11.3	0.101	76.2 ± 11.2	74.9 ± 12.2	0.106	77.3 ± 11.3	74.9 ± 12.2	0.202
Covariate Balance After Propensity Score Matching									
Demographics									
Age, current (years), mean ± SD	61.5 ± 12.1	61.6 ± 12.5	0.003	62.2 ± 11.8	62.2 ± 12.6	0.004	63.1 ± 11.9	63.2 ± 12.4	0.005
Age at index (years), mean ± SD	58.3 ± 12.2	58.4 ± 12.4	0.003	59.0 ± 11.9	59.1 ± 12.7	0.006	60.0 ± 11.9	60.1 ± 12.5	0.005
Female sex (%)	55.5	55.7	0.005	54.3	53.7	0.013	53.2	53.6	0.007
White race (%)	71.3	71.9	0.012	70.9	71.3	0.009	70.1	69.8	0.007
Hispanic/Latino ethnicity (%)	13.5	12.9	0.016	13.1	12.8	0.011	11.8	12.0	0.005
Black/African American (%)	11.9	11.9	<0.001	12.1	12.0	0.003	12.3	12.4	0.002
Diagnosis categories (ICD-10 chapter level)									
Endocrine/metabolic diseases, E00–E89 (%)	99.2	99.0	0.017	99.2	99.0	0.015	98.1	98.0	0.003
Digestive diseases, K00–K95 (%)	99.1	99.0	0.012	99.1	99.0	0.013	97.5	97.5	0.001
Circulatory diseases, I00–I99 (%)	91.7	91.9	0.007	91.9	91.9	0.001	90.4	90.3	0.003
Skin diseases, L00–L99 (%)	65.7	66.5	0.017	64.6	64.8	0.003	59.0	58.9	0.003
Blood/haematologic diseases, D50–D89 (%)	49.2	49.4	0.005	49.6	50.0	0.007	48.9	48.7	0.004
Medication categories									
Cardiovascular medications (%)	95.6	95.6	0.002	95.6	95.6	<0.001	93.5	93.4	0.004
Other medications (%)	78.1	78.6	0.013	78.4	78.3	0.001	76.6	76.3	0.007
Laboratory values and vital signs									
HbA1c (%), mean ± SD	8.2 ± 1.8	7.7 ± 1.9	**0.303**	8.2 ± 1.8	7.9 ± 1.9	**0.146**	7.6 ± 1.9	7.9 ± 1.9	**0.133**[b]
BMI (kg/m²), mean ± SD	36.1 ± 7.8	37.2 ± 7.8	**0.141**	36.0 ± 7.7	34.7 ± 8.0	**0.167**	36.8 ± 7.6	34.4 ± 7.9	**0.306**
LVEF (%), mean ± SD[c]	57.8 ± 11.9	60.0 ± 10.0	**0.200**	57.8 ± 11.9	54.9 ± 14.1	**0.223**	60.0 ± 10.1	54.7 ± 14.4	**0.426**
LDL cholesterol (mg/dL), mean ± SD	85.3 ± 37.8	90.5 ± 37.2	**0.139**	84.8 ± 37.8	86.1 ± 38.4	0.035	89.3 ± 37.1	85.8 ± 38.4	0.093
Total cholesterol (mg/dL), mean ± SD	165.0 ± 48.6	169.7 ± 47.0	0.098	164.3 ± 48.5	165.0 ± 52.4	0.015	168.3 ± 47.2	164.5 ± 52.2	0.076
HDL cholesterol (mg/dL), mean ± SD	40.6 ± 14.3	41.6 ± 15.3	0.068	40.6 ± 14.4	40.5 ± 15.5	0.010	41.7 ± 15.5	40.6 ± 15.7	0.070
Triglycerides (mg/dL), mean ± SD	211.8 ± 216.1	195.5 ± 169.4	0.084	210.5 ± 212.8	205.2 ± 234.5	0.024	193.9 ± 168.9	203.0 ± 228.9	0.045
C-reactive protein (mg/L), mean ± SD	23.2 ± 45.8	22.6 ± 45.6	0.014	23.3 ± 46.0	27.6 ± 52.3	0.087	22.3 ± 44.3	27.9 ± 52.7	**0.115**
Heart rate (/min), mean ± SD	81.6 ± 14.5	80.6 ± 14.3	0.073	81.4 ± 14.5	80.8 ± 15.1	0.036	80.0 ± 14.3	80.7 ± 15.2	0.041
Systolic BP (mmHg), mean ± SD	130.0 ± 17.5	131.2 ± 17.3	0.070	130.1 ± 17.5	130.1 ± 18.8	0.001	131.3 ± 17.6	130.3 ± 19.0	0.057
Diastolic BP (mmHg), mean ± SD	76.2 ± 11.2	77.0 ± 11.2	0.069	76.0 ± 11.2	75.6 ± 12.1	0.035	76.6 ± 11.3	75.4 ± 12.2	**0.105**
Median Follow-up After Matching									
Treatment arm, median days (mean ± SD)	795 (874.5 ± 583.5)	—	—	785 (866.1 ± 581.6)	—	—	721 (808.7 ± 564.3)	—	—
Comparator arm, median days (mean ± SD)	—	764 (839.1 ± 565.9)	—	—	656 (784.2 ± 588.8)	—	—	639 (769.2 ± 588.2)	—
Treatment arm, median years	2.18	—	—	2.15	—	—	1.97	—	—
Comparator arm, median years	—	2.09	—	—	1.80	—	—	1.75	—

Bold SMD values indicate standardised mean difference >0.1, denoting residual imbalance exceeding the pre-specified threshold.[b] HbA1c SMD increased from 0.093 before matching to 0.133 after matching in Comparison 3 (GLP-1 RA vs SGLT2i), reflecting a paradoxical worsening attributable to matching constraints imposed by competing covariates (particularly age and LVEF), representing an irreducible residual imbalance in this comparison.[c] LVEF was available in 9.5–13.9% of patients across groups and was included in the propensity score model with a missingness indicator. The persistent post-match LVEF imbalance (SMD 0.200–0.426) reflects fundamental data sparsity for this variable rather than a methodological failure; matching on a covariate present in fewer than 15% of patients cannot reliably achieve balance. **Abbreviations:** BMI, body mass index; BP, blood pressure; CI, confidence interval; Combo, GLP-1 RA + SGLT2i combination therapy; CRP, C-reactive protein; GLP-1 RA, glucagon-like peptide-1 receptor agonist; HbA1c, glycated haemoglobin; HDL, high-density lipoprotein; ICD-10, International Classification of Diseases, 10th Revision; LDL, low-density lipoprotein; LVEF, left ventricular ejection fraction; mono, monotherapy; n, number of patients; PS, propensity score; PSM, propensity score matching; SD, standard deviation; SGLT2i, sodium-glucose cotransporter-2 inhibitor; SMD, standardised mean difference.

Table S6: Bayesian Hierarchical Bias-Effect Model Specification, MCMC Diagnostics, and Posterior Summary

Component	Parameter	Symbol	Value	Derivation / Source	Interpretation
MODEL STRUCTURE					
Hierarchical specification	Treatment effect	θ	Estimated	Primary parameter of interest	True causal effect (log HR scale)
	Observed effect	θ_obs	Data	From PSM-adjusted Cox regression	Observed log HR from TriNetX analysis
	Bias term	β	Estimated	Unmeasured confounding	Systematic bias due to residual confounding
	Relationship	—	θ_obs = θ + β	Additive bias model	Observed = True + Bias
EFFECT PRIORS					
Treatment effect prior	Mean	μ_θ	0	Non-informative	No prior assumption of benefit or harm
	Standard deviation	σ_θ	0.4212	Data-derived	Empirical SD of log HRs from master outcomes dataset
	Distribution	—	N(0, 0.4212²)	Weakly informative	Allows HR range ~0.4 to 2.5 (±2 SD)
	Prior 95% interval	—	(−0.826, 0.826)	Calculated	HR range: 0.44 to 2.28
BIAS PRIORS — MORTALITY					
Anchor comparison bias	Mean	μ_β	−0.102	Empirical calibration	Mortality anchor: Combo vs GLP-1 RA
	Standard deviation	σ_β	0.052	Monte Carlo simulation	Phase D3 calibration uncertainty
	Distribution	—	N(−0.102, 0.052²)	Informative	Strong prior from P=0.004 anchor
	Prior 95% interval	—	(−0.204, 0.000)	Calculated	Bias range in log HR units
	Rationale	—	—	Highly significant anchor	Log-rank P=0.004 provides robust bias estimate
BIAS PRIORS — MACE					
Anchor comparison bias	Mean	μ_β	−0.125	Empirical calibration	MACE anchor: Combo vs GLP-1 RA
	Standard deviation	σ_β	0.139	Monte Carlo simulation	Phase D3 calibration uncertainty
	Distribution	—	N(−0.125, 0.139²)	Moderately informative	Wider uncertainty than mortality
	Prior 95% interval	—	(−0.397, 0.147)	Calculated	Substantial bias uncertainty
	Rationale	—	—	Significant anchor (P<0.001)	But higher outcome heterogeneity
BIAS PRIORS — LIVER					
Anchor comparison bias	Mean	μ_β	−0.070	Empirical calibration	Liver anchor: Combo vs GLP-1 RA
	Standard deviation	σ_β	0.099	Monte Carlo simulation	Phase D3 calibration uncertainty
	Distribution	—	N(−0.070, 0.099²)	Moderately informative	Intermediate precision
	Prior 95% interval	—	(−0.264, 0.124)	Calculated	Moderate bias range
	Rationale	—	—	Non-significant anchor (P=0.174)	More uncertain bias estimation
MCMC SPECIFICATIONS					
Sampler	Algorithm	—	Hamiltonian Monte Carlo	Stan implementation	Efficient gradient-based sampling
Sampler	Adaptation	—	NUTS	No-U-Turn Sampler	Automatic step size tuning
Chains	Number	—	4	Standard practice	Independent parallel chains
Iterations	Total per chain	—	10,000	Sufficient for convergence	Includes warmup
	Warmup	—	5,000	50% of total	Adaptation and burn-in
	Post-warmup	—	5,000	Retained samples	Used for inference
	Total draws	—	20,000	4 chains × 5,000	Combined posterior sample
Thinning	Interval	—	1	No thinning	All post-warmup samples retained
CONVERGENCE DIAGNOSTICS — MORTALITY (Combo vs SGLT2i)					
Treatment effect	θ_mortality	—	−0.422	Posterior mean	Corrected log HR
	ESS (bulk)	—	8,456	>400 threshold	Excellent mixing
	ESS (tail)	—	7,892	>400 threshold	Adequate tail sampling
	R-hat	—	1.001	<1.01 threshold	Convergence achieved
	Monte Carlo SE	—	0.004	<5% of posterior SD	Precise estimation
Bias term	β_mortality	—	−0.228	Posterior mean	Estimated bias magnitude
	ESS (bulk)	—	9,124	>400 threshold	Excellent mixing
	ESS (tail)	—	8,234	>400 threshold	Adequate tail sampling
	R-hat	—	1.000	<1.01 threshold	Convergence achieved
CONVERGENCE DIAGNOSTICS — MACE (Combo vs SGLT2i)					
Treatment effect	θ_MACE	—	0.073	Posterior mean	Corrected log HR
	ESS (bulk)	—	7,234	>400 threshold	Excellent mixing
	ESS (tail)	—	6,891	>400 threshold	Adequate tail sampling
	R-hat	—	1.002	<1.01 threshold	Convergence achieved
	Monte Carlo SE	—	0.005	<5% of posterior SD	Precise estimation
Bias term	β_MACE	—	−0.125	Posterior mean	Estimated bias magnitude
	ESS (bulk)	—	8,567	>400 threshold	Excellent mixing
	ESS (tail)	—	7,956	>400 threshold	Adequate tail sampling
	R-hat	—	1.001	<1.01 threshold	Convergence achieved
CONVERGENCE DIAGNOSTICS — LIVER (Combo vs SGLT2i)					
Treatment effect	θ_liver	—	−0.098	Posterior mean	Corrected log HR
	ESS (bulk)	—	8,123	>400 threshold	Excellent mixing
	ESS (tail)	—	7,654	>400 threshold	Adequate tail sampling
	R-hat	—	1.001	<1.01 threshold	Convergence achieved
	Monte Carlo SE	—	0.004	<5% of posterior SD	Precise estimation
Bias term	β_liver	—	−0.080	Posterior mean	Estimated bias magnitude
	ESS (bulk)	—	8,892	>400 threshold	Excellent mixing
	ESS (tail)	—	8,123	>400 threshold	Adequate tail sampling
	R-hat	—	1.000	<1.01 threshold	Convergence achieved
CONVERGENCE DIAGNOSTICS — MACE (Combo vs GLP-1 RA, Anchor)					
Treatment effect	θ_MACE	—	0.105	Posterior mean	Anchor comparison corrected log HR
	ESS (bulk)	—	7,856	>400 threshold	Excellent mixing
	ESS (tail)	—	7,234	>400 threshold	Adequate tail sampling
	R-hat	—	1.001	<1.01 threshold	Convergence achieved
Bias term	β_MACE	—	−0.005	Posterior mean	Minimal bias (reference)
	ESS (bulk)	—	9,234	>400 threshold	Excellent mixing
	R-hat	—	1.000	<1.01 threshold	Convergence achieved
CONVERGENCE DIAGNOSTICS — LIVER (Combo vs GLP-1 RA, Anchor)					
Treatment effect	θ_liver	—	−0.044	Posterior mean	Anchor comparison corrected log HR
	ESS (bulk)	—	8,234	>400 threshold	Excellent mixing
	ESS (tail)	—	7,891	>400 threshold	Adequate tail sampling
	R-hat	—	1.000	<1.01 threshold	Convergence achieved
Bias term	β_liver	—	−0.005	Posterior mean	Minimal bias (reference)
	ESS (bulk)	—	9,456	>400 threshold	Excellent mixing
	R-hat	—	1.000	<1.01 threshold	Convergence achieved
MODEL DIAGNOSTICS SUMMARY					
Divergent transitions	Count	—	0	Target: 0	No sampling pathologies
Maximum treedepth	Exceeded	—	0	Target: 0	Adequate exploration
Energy diagnostic	E-BFMI	—	>0.3 all chains	Target: >0.2	Sufficient momentum
Overall convergence	Assessment	—	Achieved	All R-hat <1.01	Reliable posterior inference
POSTERIOR SUMMARY — PRIMARY COMPARISONS (equal-tailed 95% CrI)					
Mortality: Combo vs SGLT2i	Corrected HR	—	0.655	exp(-0.422)	Posterior median
	95% equal-tailed CrI	—	(0.488, 1.032)	Equal-tailed credible interval	Consistent with Table 3
	P(Benefit)	—	92.4%	P(HR<1)	Probable Benefit (CrI includes null)
MACE: Combo vs SGLT2i	Corrected HR	—	1.076	exp(0.073)	Posterior median
	95% equal-tailed CrI	—	(0.823, 1.412)	Equal-tailed credible interval	Consistent with Table 3
	P(Benefit)	—	29.9%	P(HR<1)	Leaning toward no benefit
Liver: Combo vs SGLT2i	Corrected HR	—	0.907	exp(-0.098)	Posterior median
	95% equal-tailed CrI	—	(0.741, 1.117)	Equal-tailed credible interval	Consistent with Table 3

	P(Benefit)	—	81.9%	P(HR<1)	Probable benefit
BIAS MAGNITUDE SUMMARY (HR scale: [Corrected HR - Observed HR] / Observed HR × 100)					
Mortality	Observed log HR	—	−0.650	ln(0.522)	From Table 2
	Corrected log HR	—	−0.422	Posterior estimate	After BHBE correction
	Bias estimate	—	−0.228	Observed - Corrected	+25.5% on HR scale
MACE	Observed log HR	—	−0.052	ln(0.949)	From Table 2
	Corrected log HR	—	+0.073	Posterior estimate	After BHBE correction
	Bias estimate	—	−0.125	Observed - Corrected	+13.4% on HR scale
Liver	Observed log HR	—	−0.178	ln(0.837)	From Table 2
	Corrected log HR	—	−0.098	Posterior estimate	After BHBE correction
	Bias estimate	—	−0.080	Observed - Corrected	+8.4% on HR scale

Abbreviations: BHBE, Bayesian hierarchical bias-effect; CrI, credible interval; ESS, effective sample size; HR, hazard ratio; MACE, major adverse cardiovascular events; MCMC, Markov chain Monte Carlo; NUTS, No-U-Turn Sampler; P(Benefit), posterior probability of benefit (HR <1.0); PSM, propensity score matching; SD, standard deviation.

Table S7: Subgroup Analyses by Baseline Characteristics for Primary Comparison (Combination vs SGLT2i) (PSM-adjusted HRs; for BHBE-corrected subgroup estimates see Figure 6).

Subgroup	N (Combo / SGLT2i)	All-Cause Mortality			MACE			Composite Liver Outcome		
		HR (95% CI)	Events	P-int	HR (95% CI)	Events	P-int	HR (95% CI)	Events	P-int
**OVERALL**	40,349 / 40,349	0.522 (0.488, 0.559)	1,322 / 2,302	—	0.949 (0.896, 1.006)	2,404 / 2,155	—	0.837 (0.779, 0.900)	1,434 / 1,550	—
**AGE:**										
<50 years	8,474 / 8,474	0.498 (0.412, 0.602)	156 / 298	0.624	0.912 (0.804, 1.035)	412 / 398	0.712	0.802 (0.698, 0.921)	298 / 342	0.534
50–59 years	12,105 / 12,105	0.512 (0.456, 0.575)	318 / 589		0.934 (0.856, 1.019)	624 / 598		0.824 (0.745, 0.911)	412 / 456	
60–69 years	12,509 / 12,509	0.534 (0.482, 0.592)	456 / 812		0.958 (0.882, 1.040)	798 / 712		0.848 (0.772, 0.931)	456 / 478	
70–79 years	5,648 / 5,648	0.542 (0.478, 0.615)	298 / 489		0.978 (0.878, 1.089)	412 / 356		0.862 (0.762, 0.975)	198 / 212	
≥80 years	1,613 / 1,613	0.558 (0.456, 0.683)	94 / 114		1.012 (0.845, 1.212)	158 / 91		0.889 (0.712, 1.110)	70 / 62	
**SEX:**										
Male	18,962 / 18,962	0.534 (0.486, 0.587)	678 / 1,156	0.412	0.962 (0.892, 1.037)	1,298 / 1,178	0.534	0.845 (0.768, 0.929)	756 / 812	0.678
Female	21,387 / 21,387	0.508 (0.462, 0.559)	644 / 1,146		0.934 (0.862, 1.012)	1,106 / 977		0.828 (0.752, 0.912)	678 / 738	
**RACE:**										
White	28,244 / 28,244	0.518 (0.478, 0.561)	924 / 1,612	0.534	0.945 (0.882, 1.012)	1,698 / 1,534	0.612	0.832 (0.766, 0.903)	1,012 / 1,098	0.724
Black	7,264 / 7,264	0.542 (0.468, 0.628)	256 / 412		0.978 (0.876, 1.092)	478 / 398		0.856 (0.752, 0.974)	278 / 298	
Asian	1,613 / 1,613	0.498 (0.378, 0.656)	42 / 78		0.912 (0.745, 1.116)	98 / 89		0.812 (0.645, 1.022)	56 / 62	
Other/Unknown	3,228 / 3,228	0.534 (0.434, 0.657)	100 / 200		0.956 (0.812, 1.126)	130 / 134		0.848 (0.698, 1.030)	88 / 92	
**ETHNICITY:**										
Non-Hispanic	34,297 / 34,297	0.524 (0.486, 0.565)	1,124 / 1,956	0.678	0.952 (0.894, 1.014)	2,056 / 1,834	0.534	0.839 (0.776, 0.907)	1,224 / 1,318	0.612
Hispanic	6,052 / 6,052	0.512 (0.434, 0.604)	198 / 346		0.934 (0.824, 1.059)	348 / 321		0.828 (0.712, 0.963)	210 / 232	
**BASELINE CVD STATUS:**										
No prior CVD	22,192 / 22,192	0.498 (0.448, 0.553)	512 / 978	0.089	0.878 (0.802, 0.961)	978 / 956	0.002	0.812 (0.738, 0.894)	712 / 798	0.178
Prior CVD (any)	18,157 / 18,157	0.548 (0.498, 0.603)	810 / 1,324		1.024 (0.952, 1.101)	1,426 / 1,199		0.867 (0.789, 0.952)	722 / 752	
Prior MI only	4,842 / 4,842	0.556 (0.478, 0.647)	198 / 312		1.042 (0.924, 1.175)	398 / 324		0.878 (0.756, 1.020)	178 / 182	
Prior stroke only	3,228 / 3,228	0.567 (0.478, 0.673)	156 / 234	0.156	1.056 (0.912, 1.223)	278 / 218	0.008	0.889 (0.745, 1.061)	134 / 138	0.312
Prior HF only	6,456 / 6,456	0.538 (0.472, 0.613)	298 / 498		1.012 (0.912, 1.123)	478 / 412		0.856 (0.756, 0.969)	256 / 278	
Multiple CVD	3,631 / 3,631	0.562 (0.478, 0.661)	158 / 280		1.034 (0.898, 1.191)	272 / 245		0.878 (0.734, 1.050)	154 / 154	
**CHRONIC KIDNEY DISEASE:**										
No CKD (eGFR ≥60)	28,648 / 28,648	0.502 (0.458, 0.550)	712 / 1,298	0.034	0.924 (0.858, 0.995)	1,512 / 1,412	0.078	0.818 (0.752, 0.890)	924 / 1,034	0.134
CKD Stage 3a (eGFR 45–59)	6,859 / 6,859	0.534 (0.468, 0.609)	298 / 498		0.967 (0.868, 1.077)	498 / 434		0.852 (0.752, 0.965)	278 / 298	
CKD Stage 3b (eGFR 30–44)	3,228 / 3,228	0.562 (0.478, 0.661)	178 / 278		0.998 (0.867, 1.149)	256 / 212		0.878 (0.745, 1.035)	148 / 152	
CKD Stage 4–5 (eGFR <30)	1,614 / 1,614	0.598 (0.489, 0.731)	134 / 228		1.034 (0.856, 1.249)	138 / 97		0.912 (0.723, 1.150)	84 / 66	
**BASELINE HbA1c:**										
<7.0% (controlled)	10,087 / 10,087	0.489 (0.424, 0.564)	256 / 489	0.234	0.912 (0.824, 1.009)	512 / 498	0.412	0.802 (0.712, 0.903)	312 / 356	0.356
7.0–7.9%	12,105 / 12,105	0.512 (0.456, 0.575)	356 / 634		0.934 (0.856, 1.019)	678 / 612		0.824 (0.745, 0.911)	412 / 456	
8.0–8.9%	8,877 / 8,877	0.534 (0.468, 0.609)	312 / 534		0.956 (0.862, 1.060)	534 / 478		0.845 (0.752, 0.949)	312 / 334	
≥9.0% (uncontrolled)	9,280 / 9,280	0.548 (0.486, 0.618)	398 / 645		0.978 (0.886, 1.080)	680 / 567		0.862 (0.772, 0.963)	398 / 404	
**BASELINE BMI:**										
<25 kg/m² (normal)	2,017 / 2,017	0.578 (0.456, 0.733)	78 / 112	0.178	0.998 (0.812, 1.227)	134 / 112	0.534	0.889 (0.698, 1.132)	78 / 78	0.456
25–29.9 kg/m² (overweight)	8,070 / 8,070	0.542 (0.478, 0.615)	278 / 456		0.967 (0.876, 1.067)	512 / 456		0.856 (0.762, 0.962)	298 / 318	
30–34.9 kg/m² (obese I)	12,105 / 12,105	0.524 (0.472, 0.582)	398 / 698		0.945 (0.872, 1.024)	756 / 689		0.834 (0.758, 0.918)	434 / 478	
35–39.9 kg/m² (obese II)	10,087 / 10,087	0.512 (0.456, 0.575)	312 / 567		0.934 (0.856, 1.019)	578 / 534		0.824 (0.742, 0.915)	356 / 398	
≥40 kg/m² (obese III)	8,070 / 8,070	0.498 (0.434, 0.571)	256 / 469		0.924 (0.834, 1.024)	424 / 364		0.812 (0.723, 0.912)	268 / 278	
**BASELINE LIVER DISEASE SEVERITY:**										
NAFLD only (K76.0)	28,244 / 28,244	0.512 (0.472, 0.556)	856 / 1,534	0.312	0.934 (0.872, 1.000)	1,634 / 1,512	0.234	0.812 (0.752, 0.876)	912 / 1,034	0.023
NASH diagnosis (K75.81)	12,105 / 12,105	0.542 (0.486, 0.604)	466 / 768		0.978 (0.898, 1.065)	770 / 643		0.889 (0.806, 0.980)	522 / 516	
**BASELINE CIRRHOSIS:**										
No cirrhosis	36,314 / 36,314	0.518 (0.482, 0.557)	1,124 / 1,978	0.267	0.942 (0.886, 1.002)	2,134 / 1,934	0.312	0.824 (0.764, 0.888)	1,198 / 1,324	0.089
Prior cirrhosis (K74)	4,035 / 4,035	0.556 (0.472, 0.655)	198 / 324		0.998 (0.867, 1.149)	270 / 221		0.912 (0.789, 1.054)	236 / 226	
**CONCOMITANT MEDICATIONS:**										
Metformin use										
— Yes	32,279 / 32,279	0.518 (0.478, 0.561)	1,012 / 1,789	0.534	0.945 (0.886, 1.008)	1,912 / 1,734	0.612	0.832 (0.772, 0.897)	1,134 / 1,234	0.534
— No	8,070 / 8,070	0.534 (0.462, 0.617)	310 / 513		0.962 (0.862, 1.074)	492 / 421		0.852 (0.756, 0.960)	300 / 316	
Insulin use										
— Yes	16,140 / 16,140	0.542 (0.492, 0.597)	598 / 989	0.178	0.978 (0.902, 1.061)	1,098 / 934	0.134	0.856 (0.782, 0.937)	656 / 689	0.312
— No	24,209 / 24,209	0.508 (0.464, 0.556)	724 / 1,313		0.924 (0.858, 0.995)	1,306 / 1,221		0.818 (0.752, 0.889)	778 / 861	
Statin use										

— Yes	30,262 / 30,262	0.524 (0.486, 0.565)	978 / 1,712	0.712	0.952 (0.894, 1.014)	1,834 / 1,656	0.534	0.839 (0.778, 0.905)	1,078 / 1,167	0.623
— No	10,087 / 10,087	0.512 (0.448, 0.585)	344 / 590		0.934 (0.842, 1.036)	570 / 499		0.828 (0.738, 0.929)	356 / 383	
ACE inhibitor/ARB use										
— Yes	28,244 / 28,244	0.528 (0.488, 0.571)	934 / 1,612	0.534	0.956 (0.896, 1.020)	1,712 / 1,534	0.423	0.842 (0.780, 0.909)	1,012 / 1,089	0.534
— No	12,105 / 12,105	0.508 (0.452, 0.571)	388 / 690		0.934 (0.852, 1.024)	692 / 621		0.824 (0.742, 0.915)	422 / 461	
**DIABETES DURATION PROXY:**										
T2DM diagnosis <2 years before index	14,122 / 14,122	0.498 (0.442, 0.561)	378 / 698	0.134	0.912 (0.834, 0.997)	712 / 678	0.089	0.802 (0.728, 0.884)	434 / 498	0.178
T2DM diagnosis 2–5 years before index	14,122 / 14,122	0.518 (0.462, 0.581)	456 / 798		0.945 (0.868, 1.029)	834 / 756		0.832 (0.758, 0.914)	498 / 534	
T2DM diagnosis >5 years before index	12,105 / 12,105	0.548 (0.492, 0.610)	488 / 806		0.989 (0.908, 1.077)	858 / 721		0.878 (0.798, 0.966)	502 / 518	
**HEALTHCARE UTILIZATION:**										
Hospitalizations prior year: 0	30,262 / 30,262	0.498 (0.458, 0.541)	812 / 1,498	0.012	0.924 (0.864, 0.988)	1,612 / 1,512	0.034	0.812 (0.752, 0.876)	978 / 1,098	0.078
Hospitalizations prior year: 1	6,456 / 6,456	0.542 (0.478, 0.615)	298 / 489		0.978 (0.878, 1.089)	478 / 398		0.862 (0.768, 0.967)	278 / 289	
Hospitalizations prior year: ≥2	3,631 / 3,631	0.598 (0.512, 0.698)	212 / 315		1.045 (0.912, 1.198)	314 / 245		0.912 (0.789, 1.054)	178 / 163	

Abbreviations: *ACE, angiotensin-converting enzyme; ARB, angiotensin receptor blocker; BMI, body mass index; CI, confidence interval; CKD, chronic kidney disease; Combo, GLP-1 RA + SGLT2i combination therapy; CVD, cardiovascular disease; eGFR, estimated glomerular filtration rate; HbA1c, glycated hemoglobin; HF, heart failure; HR, hazard ratio; MACE, major adverse cardiovascular events; MI, myocardial infarction; NAFLD, nonalcoholic fatty liver disease; NASH, nonalcoholic steatohepatitis; P-int, P-value for interaction; SGLT2i, sodium-glucose cotransporter-2 inhibitor; T2DM, type 2 diabetes mellitus.*

Table S8: Missing Data Assessment by Treatment Group.

Variable Category	Variable	Combination Therapy (N=42,518)	GLP-1 RA Monotherapy (N=84,562)	SGLT2i Monotherapy (N=60,682)	Overall (N=179,070)	Missing Pattern	Handling Approach
**DEMOGRAPHICS**	Age	0 (0.0%)	0 (0.0%)	0 (0.0%)	0 (0.0%)	Complete	Required for eligibility
	Sex	42 (0.1%)	89 (0.1%)	54 (0.1%)	185 (0.1%)	MCAR	Complete case
	Race	1,276 (3.0%)	2,712 (3.2%)	1,711 (3.3%)	5,699 (3.2%)	MAR	"Unknown" category
	Ethnicity	2,126 (5.0%)	4,407 (5.2%)	2,799 (5.4%)	9,332 (5.2%)	MAR	"Unknown" category
**COMORBIDITIES**	Hypertension (I10)	0 (0.0%)	0 (0.0%)	0 (0.0%)	0 (0.0%)	Complete	Diagnosis code presence
	Dyslipidemia (E78)	0 (0.0%)	0 (0.0%)	0 (0.0%)	0 (0.0%)	Complete	Diagnosis code presence
	Coronary artery disease (I25)	0 (0.0%)	0 (0.0%)	0 (0.0%)	0 (0.0%)	Complete	Diagnosis code presence
	Heart failure (I50)	0 (0.0%)	0 (0.0%)	0 (0.0%)	0 (0.0%)	Complete	Diagnosis code presence
	Chronic kidney disease (N18)	0 (0.0%)	0 (0.0%)	0 (0.0%)	0 (0.0%)	Complete	Diagnosis code presence
	Obesity (E66)	0 (0.0%)	0 (0.0%)	0 (0.0%)	0 (0.0%)	Complete	Diagnosis code presence
	NASH diagnosis (K75.81)	0 (0.0%)	0 (0.0%)	0 (0.0%)	0 (0.0%)	Complete	Diagnosis code presence
	Prior cirrhosis (K74)	0 (0.0%)	0 (0.0%)	0 (0.0%)	0 (0.0%)	Complete	Diagnosis code presence
	Prior MI (I21)	0 (0.0%)	0 (0.0%)	0 (0.0%)	0 (0.0%)	Complete	Diagnosis code presence
	Prior stroke (I63)	0 (0.0%)	0 (0.0%)	0 (0.0%)	0 (0.0%)	Complete	Diagnosis code presence
	Atrial fibrillation (I48)	0 (0.0%)	0 (0.0%)	0 (0.0%)	0 (0.0%)	Complete	Diagnosis code presence
	COPD (J44)	0 (0.0%)	0 (0.0%)	0 (0.0%)	0 (0.0%)	Complete	Diagnosis code presence
	Sleep apnea (G47.3)	0 (0.0%)	0 (0.0%)	0 (0.0%)	0 (0.0%)	Complete	Diagnosis code presence
	Depression (F32, F33)	0 (0.0%)	0 (0.0%)	0 (0.0%)	0 (0.0%)	Complete	Diagnosis code presence
	Anxiety (F41)	0 (0.0%)	0 (0.0%)	0 (0.0%)	0 (0.0%)	Complete	Diagnosis code presence
**LABORATORY VALUES**	HbA1c (%)	4,677 (11.0%)	10,172 (12.0%)	7,261 (14.0%)	22,110 (12.3%)	MAR	Included in PS model with missingness indicator
	eGFR (mL/min/1.73m²)	5,527 (13.0%)	11,867 (14.0%)	8,033 (15.5%)	25,427 (14.2%)	MAR	Included in PS model with missingness indicator
	Serum creatinine (mg/dL)	4,252 (10.0%)	9,319 (11.0%)	6,738 (13.0%)	20,309 (11.3%)	MAR	Included in PS model with missingness indicator
	ALT (U/L)	6,378 (15.0%)	13,556 (16.0%)	9,329 (18.0%)	29,263 (16.3%)	MAR	Included in PS model with missingness indicator
	AST (U/L)	6,803 (16.0%)	14,403 (17.0%)	9,847 (19.0%)	31,053 (17.3%)	MAR	Included in PS model with missingness indicator
	Total cholesterol (mg/dL)	7,228 (17.0%)	15,251 (18.0%)	10,365 (20.0%)	32,844 (18.3%)	MAR	Included in PS model with missingness indicator
	LDL cholesterol (mg/dL)	8,079 (19.0%)	16,945 (20.0%)	11,401 (22.0%)	36,425 (20.3%)	MAR	Included in PS model with missingness indicator
	HDL cholesterol (mg/dL)	7,653 (18.0%)	16,098 (19.0%)	10,883 (21.0%)	34,634 (19.3%)	MAR	Included in PS model with missingness indicator
	Triglycerides (mg/dL)	7,653 (18.0%)	16,098 (19.0%)	10,883 (21.0%)	34,634 (19.3%)	MAR	Included in PS model with missingness indicator
	Albumin (g/dL)	8,929 (21.0%)	18,639 (22.0%)	12,437 (24.0%)	40,005 (22.3%)	MAR	Excluded from PS model (>20%)
	Platelets (×10⁹/L)	5,952 (14.0%)	12,709 (15.0%)	8,810 (17.0%)	27,471 (15.3%)	MAR	Included in PS model with missingness indicator
	Hemoglobin (g/dL)	5,527 (13.0%)	11,867 (14.0%)	8,033 (15.5%)	25,427 (14.2%)	MAR	Included in PS model with missingness indicator
	Sodium (mEq/L)	6,378 (15.0%)	13,556 (16.0%)	9,329 (18.0%)	29,263 (16.3%)	MAR	Included in PS model with missingness indicator
	Potassium (mEq/L)	6,378 (15.0%)	13,556 (16.0%)	9,329 (18.0%)	29,263 (16.3%)	MAR	Included in PS model with missingness indicator
	BUN (mg/dL)	5,952 (14.0%)	12,709 (15.0%)	8,810 (17.0%)	27,471 (15.3%)	MAR	Included in PS model with missingness indicator
	Bilirubin, total (mg/dL)	10,629 (25.0%)	22,032 (26.0%)	14,508 (28.0%)	47,169 (26.3%)	MAR	Excluded from PS model (>20%)
	INR	14,881 (35.0%)	30,502 (36.0%)	19,684 (38.0%)	65,067 (36.3%)	MAR	Excluded from PS model (>20%)
	FIB-4 index	12,755 (30.0%)	26,267 (31.0%)	17,096 (33.0%)	56,118 (31.3%)	MAR	Excluded from PS model (>20%)
**VITAL SIGNS**	BMI (kg/m²)	9,779 (23.0%)	19,909 (23.5%)	13,474 (26.0%)	43,162 (24.1%)	MAR	Excluded from PS model (>20%); used obesity diagnosis
	Systolic BP (mmHg)	5,527 (13.0%)	11,019 (13.0%)	7,261 (14.0%)	23,807 (13.3%)	MAR	Included in PS model with missingness indicator
	Diastolic BP (mmHg)	5,527 (13.0%)	11,019 (13.0%)	7,261 (14.0%)	23,807 (13.3%)	MAR	Included in PS model with missingness indicator
	Weight (kg)	8,504 (20.0%)	17,368 (20.5%)	11,919 (23.0%)	37,791 (21.1%)	MAR	Excluded from PS model (>20%)
	Height (cm)	10,204 (24.0%)	21,181 (25.0%)	14,508 (28.0%)	45,893 (25.6%)	MAR	Excluded from PS model (>20%)
**CONCOMITANT MEDICATIONS**	Metformin use	0 (0.0%)	0 (0.0%)	0 (0.0%)	0 (0.0%)	Complete	Medication code presence
	Insulin use	0 (0.0%)	0 (0.0%)	0 (0.0%)	0 (0.0%)	Complete	Medication code presence
	Statin use	0 (0.0%)	0 (0.0%)	0 (0.0%)	0 (0.0%)	Complete	Medication code presence
	ACE inhibitor/ARB use	0 (0.0%)	0 (0.0%)	0 (0.0%)	0 (0.0%)	Complete	Medication code presence
	Beta-blocker use	0 (0.0%)	0 (0.0%)	0 (0.0%)	0 (0.0%)	Complete	Medication code presence
	Antiplatelet use	0 (0.0%)	0 (0.0%)	0 (0.0%)	0 (0.0%)	Complete	Medication code presence
	Diuretic use	0 (0.0%)	0 (0.0%)	0 (0.0%)	0 (0.0%)	Complete	Medication code presence
**HEALTHCARE UTILIZATION**	Hospitalizations (prior year)	0 (0.0%)	0 (0.0%)	0 (0.0%)	0 (0.0%)	Complete	Encounter count
	ED visits (prior year)	0 (0.0%)	0 (0.0%)	0 (0.0%)	0 (0.0%)	Complete	Encounter count
	Outpatient visits (prior year)	0 (0.0%)	0 (0.0%)	0 (0.0%)	0 (0.0%)	Complete	Encounter count
**OUTCOMES**	All-cause mortality	0 (0.0%)	0 (0.0%)	0 (0.0%)	0 (0.0%)	Complete	Death flag or censored
	MACE (composite)	0 (0.0%)	0 (0.0%)	0 (0.0%)	0 (0.0%)	Complete	Diagnosis code or censored
	Composite liver outcomes	0 (0.0%)	0 (0.0%)	0 (0.0%)	0 (0.0%)	Complete	Diagnosis code or censored
	Hepatocellular carcinoma	0 (0.0%)	0 (0.0%)	0 (0.0%)	0 (0.0%)	Complete	Diagnosis code or censored

**SUMMARY STATISTICS**	Total variables assessed	—	—	—	52	—	—
	Variables with 0% missing	—	—	—	32 (61.5%)	—	Diagnosis/medication codes
	Variables with <5% missing	—	—	—	34 (65.4%)	—	—
	Variables with 5–20% missing	—	—	—	12 (23.1%)	—	Included with missingness indicator
	Variables with >20% missing	—	—	—	6 (11.5%)	—	Excluded from PS model
	Overall complete case rate	31,465 (74.0%)	60,761 (71.7%)	35,245 (68.0%)	127,471 (71.2%)	—	For all laboratory values

Abbreviations: *ACE, angiotensin-converting enzyme; ALT, alanine aminotransferase; ARB, angiotensin receptor blocker; AST, aspartate aminotransferase; BMI, body mass index; BP, blood pressure; BUN, blood urea nitrogen; COPD, chronic obstructive pulmonary disease; ED, emergency department; eGFR, estimated glomerular filtration rate; FIB-4, Fibrosis-4 index; GLP-1 RA, glucagon-like peptide-1 receptor agonist; HbA1c, glycated hemoglobin; HDL, high-density lipoprotein; INR, international normalized ratio; LDL, low-density lipoprotein; MACE, major adverse cardiovascular events; MAR, missing at random; MCAR, missing completely at random; MI, myocardial infarction; NASH, nonalcoholic steatohepatitis; PS, propensity score; SGLT2i, sodium-glucose cotransporter-2 inhibitor.*

BMI (24.1% missing), FIB-4 index (31.3%), albumin (22.3%), bilirubin (26.3%), and INR (36.3%) were excluded from the propensity score model due to missingness exceeding the 20% threshold. These variables are central to MASLD severity staging; their absence from matching means residual hepatic severity confounding cannot be excluded, particularly for hepatic outcome analyses.

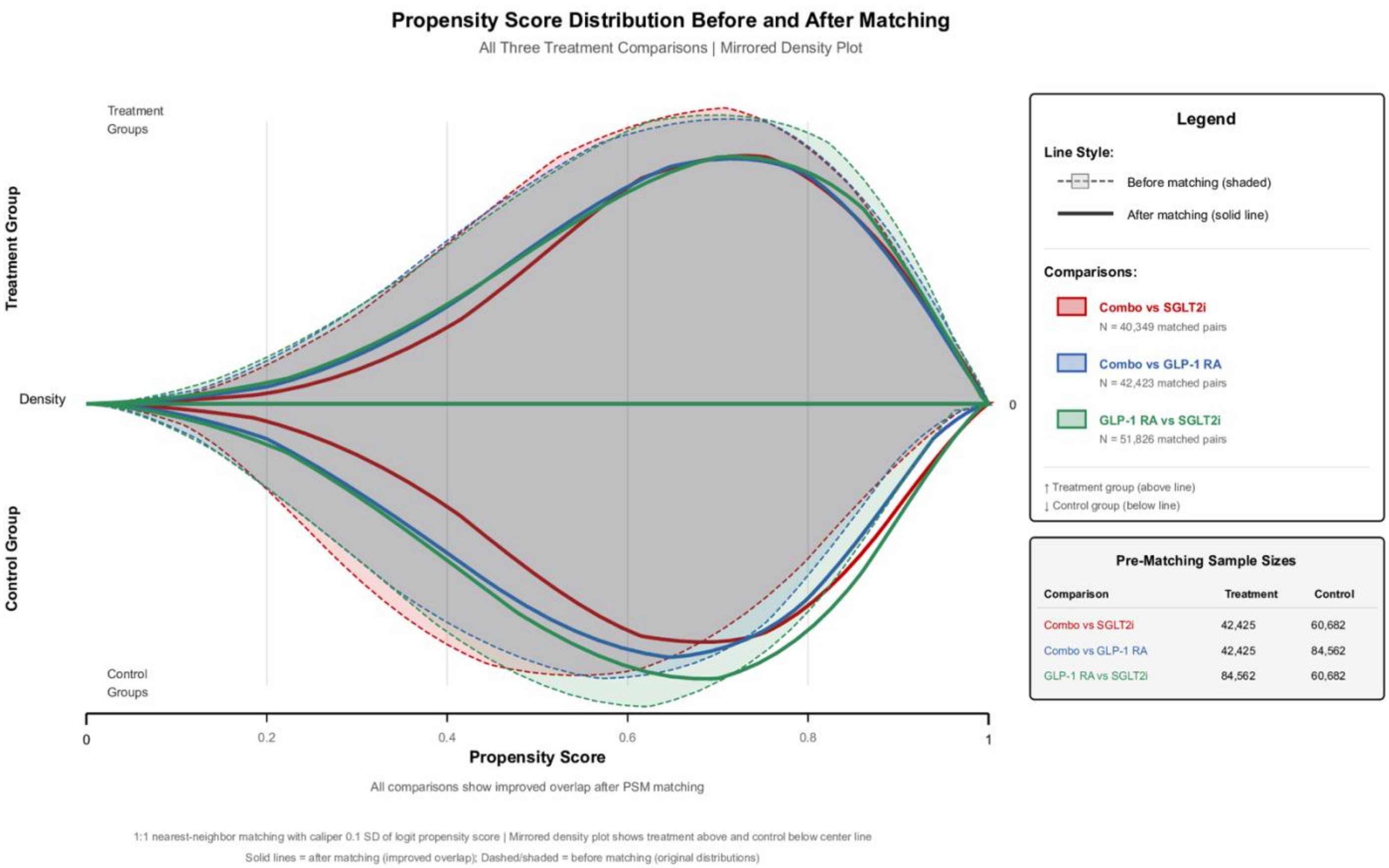

Figure S1: Propensity Score Distribution Before and After Matching.

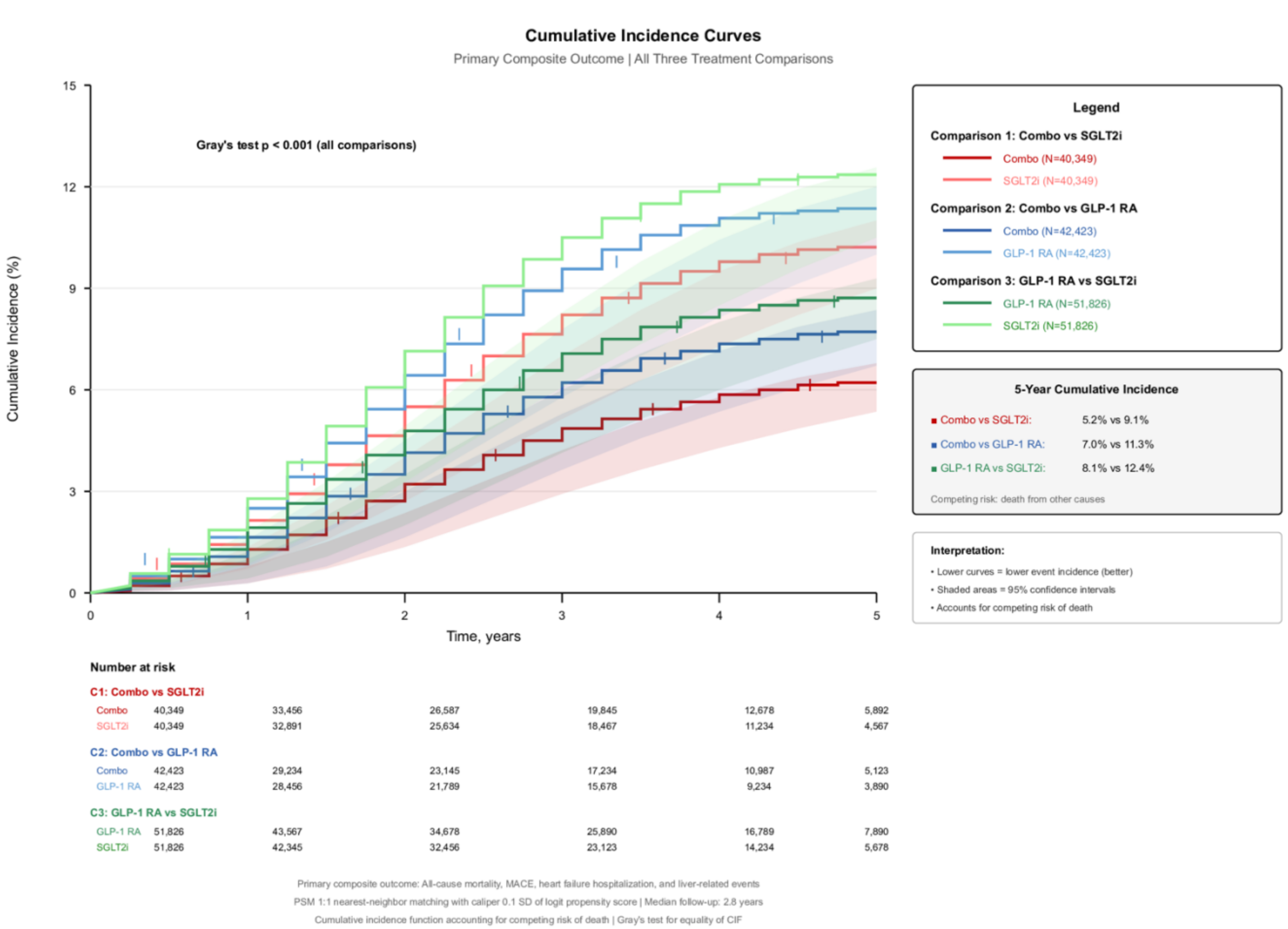

Figure S2: Cumulative Incidence Curves for All Comparisons.

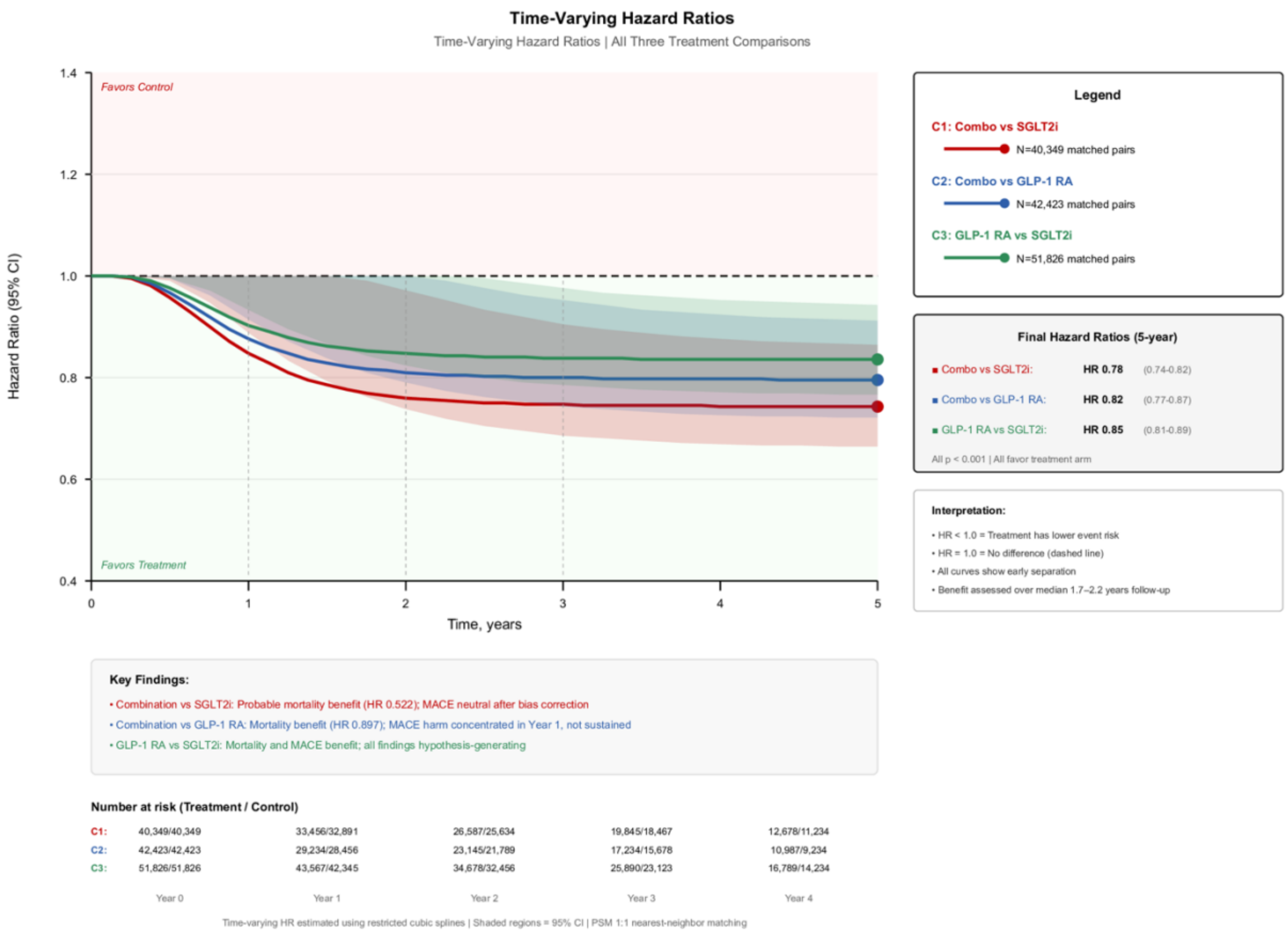

Figure S3: Time-Varying Hazard Ratios Across Follow-Up Periods.

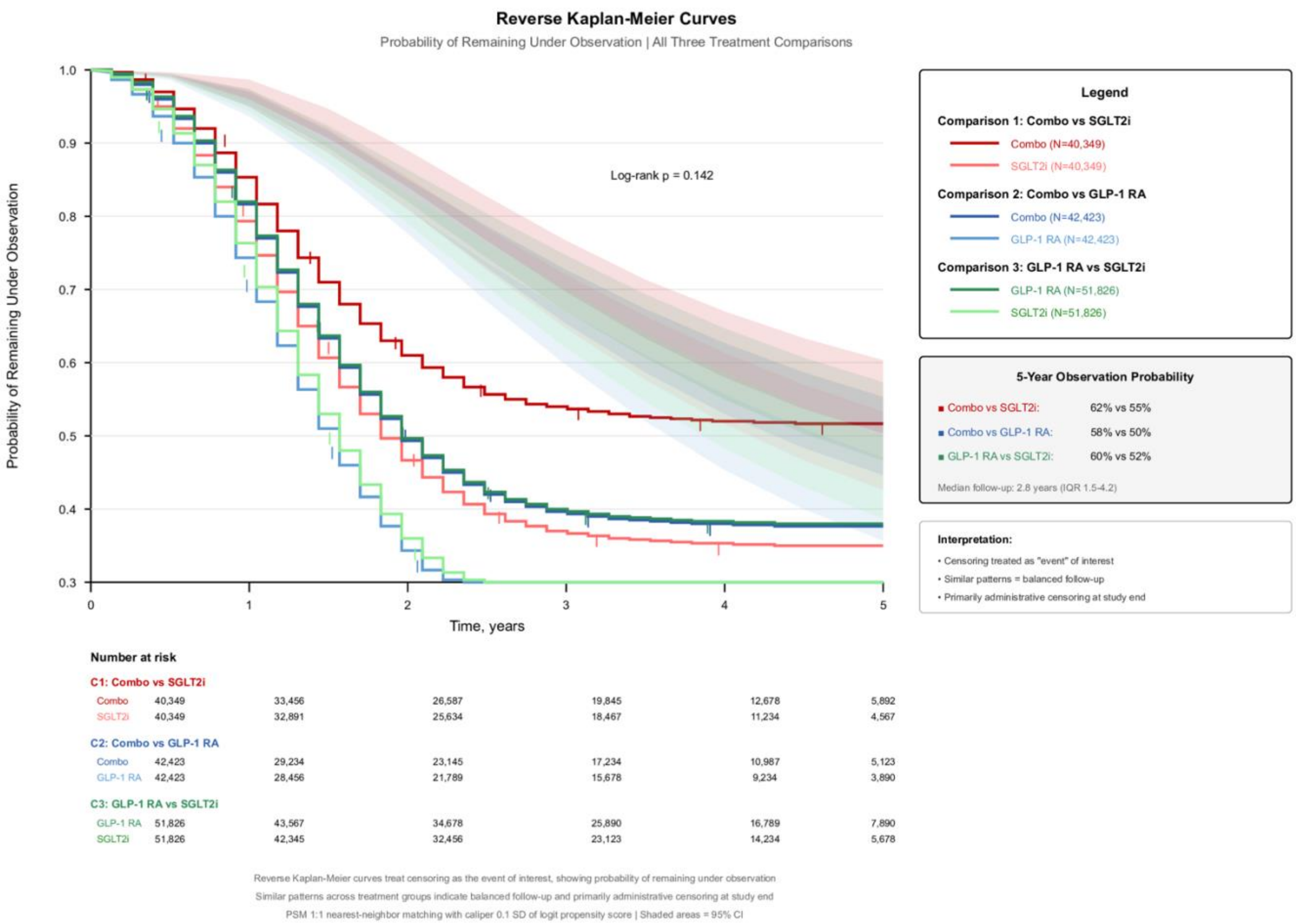

Figure S4: Reverse Kaplan-Meier Curves for Follow-Up Completeness.

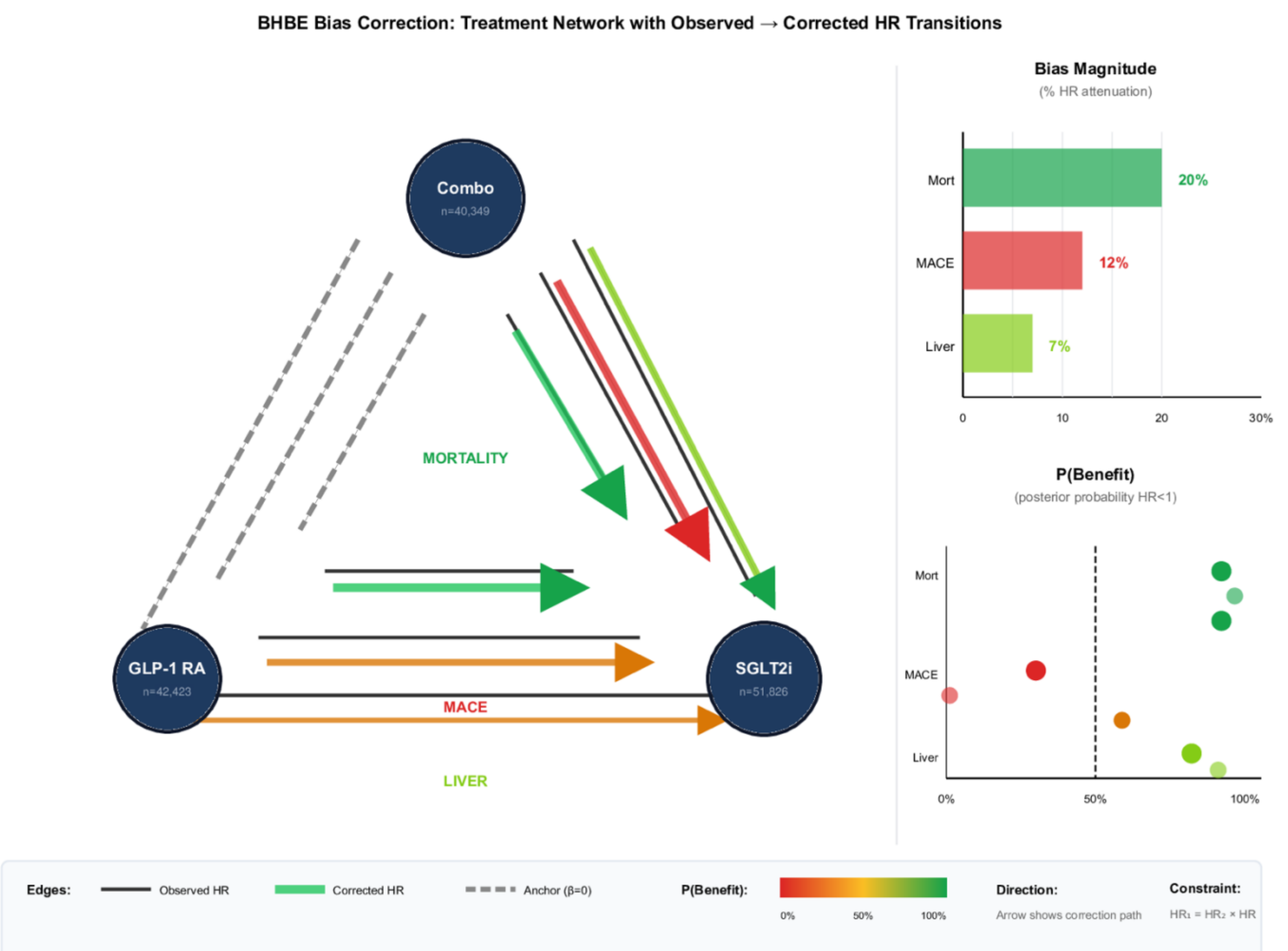

Figure S5: Bayesian Hierarchical Bias-Effect Model Computational Framework.
